# The anchor design of anchor-based method to determine the minimal clinically important difference: a systematic review

**DOI:** 10.1186/s12955-023-02157-3

**Published:** 2023-07-15

**Authors:** Yu Zhang, Xiaoyu Xi, Yuankai Huang

**Affiliations:** China Pharmaceutical University, No. 639, Longmian Avenue, Jiangning District, Nanjing, 211198 Jiangsu Province China

**Keywords:** Minimal clinically important difference, Anchor-based method, Patient-reported Outcome

## Abstract

**Background:**

Positive results for clinical outcomes should be not only statistically significant, but also clinically significant. The minimum clinically important difference (MCID) is used to define the minimum threshold of clinical significance. The anchor-based method is a classical method for ascertaining MCID. This study aimed to summarise the design of the anchors of the anchor-based method by reviewing the existing research and providing references and suggestions.

**Method:**

This study was mainly based on literature research. We performed a systematic search using Web of Science, PubMed, CNKI, Wanfang, and VIP databases. Two reviewers independently screened titles and abstracts to identify relevant articles. Data were extracted from eligible articles using a predefined data collection form. Discrepancies were resolved by discussion and the involvement of a third reviewer.

**Result:**

Three hundred and forty articles were retained for final analysis. For the design of anchors, Subjective anchors (99.12%) were the most common type of anchor used, mainly the Patient’s rating of change or patient satisfaction (66.47%) and related scale health status evaluation items or scores (39.41%). Almost half of the studies (48.53%) did not assess the correlation test between the anchor and the research indicator or scale. The cut-off values and grouping were usually based on the choice of the anchor types. In addition, due to the large number of included studies, this study selected the most calculated SF-36 (28 articles) for an in-depth analysis. The results showed that the overall design of the anchor and the cut-off value were the same as above. The statistical methods used were mostly traditional (mean change, ROC). The MCID thresholds of these studies had a wide range (SF-36 PCS: 2–17.4, SF-36 MCS: 1.46–10.28), and different anchors or statistical methods lead to different results.

**Conclusion:**

It is of great importance to select several types of anchors and to use more reliable statistical methods to calculate the MCID. It is suggested that the order of selection of anchors should be: objective anchors > anchors with established MCID in subjective anchors (specific scale > generic scale) > ranked anchors in subjective anchors. The selection of internal anchors should be avoided, and anchors should be evaluated by a correlation test.

**Supplementary Information:**

The online version contains supplementary material available at 10.1186/s12955-023-02157-3.

## Introduction

In 1989, Jaeschke and Guyatt formally defined MCID as the smallest difference in score in the domain of interest which patients perceive as beneficial and which would mandate, in the absence of troublesome side effects and excessive cost, a change in the patient’s management [1]. MCID can provide the basis for judging the clinical significance and clinical decision making of test results [2]. MCID was first proposed to better explain the clinical significance of the changes in the scores of patient-reported outcomes, such as the Quality of Life Scale. Later, the application of MCID was gradually extended to the 6 min walk test (6MWT) [3]、the fall rate [4]、the troponin [5], bridging the gap between statistics and clinical practice.

Currently, the commonly used MCID calculation methods include anchor-based method, distribution-based method, literature analysis method and expert consensus method. The anchor method is also called “external reference” method, that is, one anchor is selected as the external indicator and examines the relationship between scores on the instrument whose interpretation is under question (the target instrument) and some independent measure (an anchor) [6]. The distribution-based method is to ascertain MCID based on the distribution of sample data. Common indicators include 0.5SD, SEM, etc., but the estimation results obtained from a statistical perspective alone cannot scientifically explain the MCID. The literature analysis systematically reviews the published literature and synthesizes the results as the reference basis for MCID [7]. Obviously, it relies on the secondary literature, which should only play an auxiliary role in determining MCID. The expert consensus method is based on the group decision and consensus method to determine MCID [8], but the results only rely on the subjective judgment of experts and are also only used as an auxiliary method. Therefore, the anchor-based method is generally the preferred method for ascertaining the MCID. When it is difficult to find a suitable anchor, the distribution-based method is adopted, the distribution-based method is also commonly used. The literature analysis method and the expert consensus method are relatively niche approaches.

For the selection of anchors, the existing anchors can be divided into subjective anchors and objective anchors. A subjective anchor is a judgment about the changes in the disease in the past period, which is prone to multiple biases. The objective anchor can select laboratory examination indicators, physiological examination indicators and clinical outcomes [9]. However, whether the anchor selection is appropriate still depends on the correlation test between the anchor and the change in the scores on the research indicators or scale after the investigation, that is, the selected external anchor and the target measurement instrument should have a moderate correlation, and the correlation coefficient recommended by Revicki should be > 0.3–0.35 [7]. In addition, although recent studies have shown that the reliability and the degree of current state bias for the selected anchor can be assessed, it is still used as a subsequent verification method and is only limited to the transition ratings [10]. There is still a lack of research on anchor selection strategies. At the same time, after selecting the appropriate anchor, the patients need to be grouped according to the anchor cut-off value, which is the threshold value for dividing the patients into slight change groups and unchanged groups according to the anchor. However, the determination of the anchor cut-off value has not reached a consensus. For the statistical methods, traditional methods include mean change (within or between groups), ROC curve method, and linear regression method. In recent years, these methods have been criticized [11] and we have seen the emergence of a new method: the adjusted predictive modelling method [12], which is more reliable.

At present, there are a large number of articles that use the anchor-based method to calculate MCID. But for the anchor-based method, more attention is paid to the improvement of the statistical method, few people pay attention to the selection of anchors, which plays an important role in the calculation of MCID. Since there is no research on the anchor design of anchor-based method to calculate MCID, this study focused on the anchor design and aimed to summarise the anchor design rules in the existing research, so as to provide references for the standardised calculation of MCID by anchor-based method from a new perspective.

## Method

### Search and selection strategy

There are numerous articles in the field of MCID. We preliminarily searched for relevant research on MCID with “minimal clinically important difference” and found that research on calculating MCID generally clearly mentions MCID and the calculation method used (such as anchor-based or distribution-based method) in the title or abstract. In terms of setting search terms, we attempted various combinations and ultimately found that the articles retrieved using “minimal clinically important difference” and “anchor” as search terms best met our requirements. On this basis, the Chinese search terms take into account the differences in translation, and all use the subject term retrieval. Finally, Web of Science, PubMed, CNKI, Wanfang and VIP databases were retrieved, with the retrieval time limit from 2000 to June 2022. The following search strategy was used (taking PubMed as an example): (minimal clinically important difference[Title/Abstract]) AND (anchor[Title/Abstract]). The inclusion criteria: ①MCID was calculated by anchor-based method; ②The selected anchors and cut-off values were illustrated; ③The calculation process and results were relatively complete. The exclusion criteria: ①Repetitive literature; ② Irrelevant literature; ③Literature reviews; ④MCID was not calculated by anchor-based; ⑤Full text cannot be obtained.

### Data extraction

Two reviewers independently screened titles and abstracts to identify relevant articles. Data were extracted from eligible articles using a predefined data collection form. Discrepancies were resolved by discussion and the involvement of a third reviewer. This study focused on the anchor design and specific statistical process (including the determination of cut-off value and the selection of statistical methods) in the existing research. Therefore, the final content of literature information extraction mainly includes: ①Basic information included in the study, including the first author, publication time, research purpose and disease field; ②Research indicators or scales; ③Anchor design: anchor selection and the determination of cut-off value and grouping.

### Data analysis

Firstly, all studies were classified according to the anchor selection (subjective anchor or objective anchor). Since there were many types of subjective anchors, but there was no classification standard for subjective anchor types at present, based on the types of subjective anchors in existing research, divides them into two categories: ①Health status evaluation items or scores in the relevant scales of research indicators, such as the Health Transition Item of the SF-36(“In general, would you say your health is: excellent, very good, good, fair, or poor?”) and the scores of St George's Respiratory Questionnaire (SGRQ). ②Patient’s ratings of change or patient satisfaction, such as the Global Rating of Change Scale (GROC) and the Clinical Global Impression (CGI). Secondly, we summarised the determination of cut-offs and groupings according to different anchors. Finally, we selected one scale for further analysis.

## Result

**Fig. 1:**
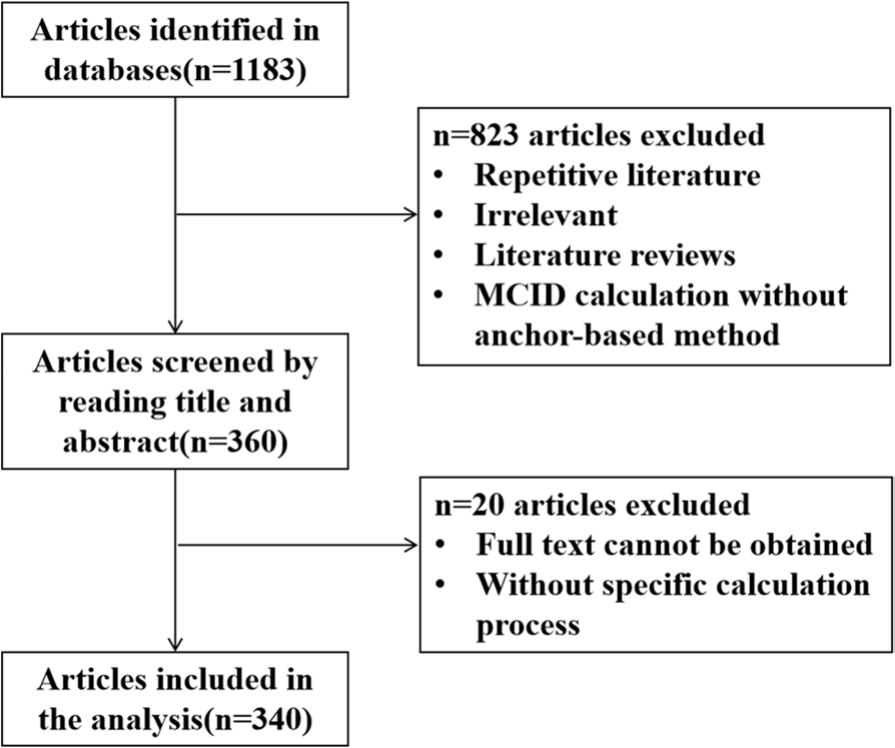
Flow chart of the study selection procedure

The literature screening process is shown in Fig. 1. This study finally included and summarised 340 literatures on anchor-based method of calculating MCID (Appendix 1).

Table 1 shows the basic information of the included literature. The existing research has covered many disease fields, mainly focusing on orthopedic diseases (40.29%), nervous system diseases (14.41%), respiratory system diseases (12.35%) and cancer fields (7.06%). The research indicators are mainly patient-reported outcomes (94.12%).

**Table 1:** Basic information of included study (*N* = 340)

	N	Percent
**Disease field**		
Orthopedic	137	40.29%
Neuropathy	49	14.41%
Respiratory	42	12.35%
Cancer	24	7.06%
Immune	12	3.53%
Mental	11	3.24%
Cardiovascular	10	2.94%
Digestive	10	2.94%
Reproductive	7	2.58%
Eye	5	1.47%
Skin	5	1.47%
Others	28	8.24%
**Research indicator or scale**^**a**^		
Patient-reported outcome(PRO)	320	94.12%
Clinical objective indicator	25	7.35%
**Number of anchors**		
1	249	73.24%
> 1	91	26.76%
**Anchor**^**b**^		
Subjective anchor	337	99.12%
Items of health status evaluation changes or scores in the scale related to research indicators	134	39.41%
Generic scale	40	11.76%
Special scale	94	27.65%
The patient's clinical change rating or patient satisfaction	226	66.47%
Objective anchor	20	5.88%
**Correlation test between anchor and research scale/index**		
Yes	175	51.47%
No	165	48.53%
**Number of statistical methods**		
1	261	76.76%
> 1	79	23.24%

^a b^ Some studies calculate MCID for multiple research scales or indicators at the same time, and use multiple anchors, the percentage of two choices of research scales/indicators and anchor types is not 100%

For the design of the anchor, most studies used a single anchor (73.24%) to calculate MCID, and some studies used multiple anchors (26.76%). The type of anchor selected was mainly the subjective anchor (99.12%), it mainly includes: ①Items of health status evaluation or scores in the scale related to research indicators or scales (39.41%). There mainly were specific scale② The patient’s rating of change or patient satisfaction (66.47%). The articles using objective anchors (5.58%) are rarely used, and most of them use subjective anchors together to calculate MCID. In addition, nearly half (48.53%) of the included studies did not evaluate correlation tests between anchors and research indicators and scales.

The determination of the anchor cutoff value and grouping is shown in Table 2 (according to the situation of most studies summarised in the included study). The determination of the cut-off value and grouping was related to the selected anchor. For the anchor with MCID established, the MCID of the anchor was the cut-off value; for rank anchors, 5, 7 or 15 point Likert scales were most commonly used, and certain grade options were selected as the cut-off value.

**Table 2:** Determination of anchor cutoff value and grouping ^c^

Anchor (N, Percent)		Option level of anchor(N, Percent)	Determination of cut-off values and grouping
Anchor with MCID established(110,32.35%)	①Scores in the scale related to research indicators related scales in subjective anchors (90, 26.47%)		Patients were divided into a minimal change group and an unchanged group according to anchors
	②Objective indicator(20, 5.88%)		
Rank anchor(270, 79.41%)	①Items of health status evaluation in the scale related to research indicators in subjective anchors(44, 12.94%) ②Patient’s rating of change or patient satisfaction in subjective anchors(226, 66.47%)	5-point Likert (117, 34.41%): 1 = much worse; 2 = a little worse; 3 = no change; 4 = a little better; 5 = much better	minimal improvement group: 2 minimal deterioration group: 4 unchanged group: 3
		7-point Likert (65, 19.12%): 1 = very much worse; 2 = much worse; 3 = a little worse, 4 = no change; 5 = a little better; 6 = much better; and 7 = very much better	minimal improvement group:5 or 6 minimal deterioration group: 2 or 3 unchanged group: 4
		15-point Likert (32,9.41%): -7-A very great deal worse -6-A great deal worse -5-A good deal worse -4-Moderately worse -3-Somewhat worse -2-A little worse -1-Almost the same, hardly any worse at all 0-No change +1-Almost the same, hardly any better at all +2-A little better +3-Somewhat better +4-Moderately better +5-A good deal better +6-A great deal better +7-A very great deal better	minimal improvement group:-2,-3 minimal deterioration group: + 2, + 3 unchanged group: -1, 0, + 1

^C^ Some studies calculate MCID for multiple research scales or indicators at the same time, and use multiple anchors, the percentage of anchor types is not 100%

Due to the large number of studies included, only the SF-36 (*n* = 28) with the highest number of calculations was selected for detailed analysis. Table 3 shows the design of the anchors for the SF-36. For the design of the anchor, the most commonly used anchor is the patient’s change rating of change with 5, 7 or 15 point Likert scales (*n* = 14), followed by the Healthy Transition Item of SF-36 (*n* = 9), and finally the related scale scores (*n* = 5). More than half of the articles (*n* = 15) did not test the correlation between the anchor and the SF-36 scores. The determination of cut-off values and grouping mostly conformed to the conditions summarised in Table 2, but two articles were special, they were anchored by SF-36 scores and its physician function scores, and the results of their statistical distribution were taken as cut-off values.

**Table 3:** The design of anchors for SF-36 (*n* = 28)

Author(Year)	Disease	Anchor	Correlation test	Statistical methods	MCID threshold values	
					**PCS**	**MCS**
Angst, F.(2001) [13]	Osteoarthritis of the lower extremities	Patient’s rating of change (5-point Likert scales)	No	Mean change (between groups)	2	-
Escobar, A(2005) [14]	Total knee replacement (TKR)	Patient’s rating of change (5-point Likert scales)	No	Mean change (within group)	-	-
Bilbao, A.(2009) [15]	Cataract	Patient’s rating of change (5-point Likert scales)	No	Mean change (within group)	-	-
Coteur, G.(2009) [16]	Active Crohn's disease	①Crohn’s Disease Activity Index (CDAI) ②Inflammatory Bowel Disease Questionnaire (IBDQ)	Yes	Linear regression	**CDAI:3.6** **IBDQ:4.1**	**CDAI:4.1** **IBDQ:3.9**
Carreon, L.Y.(2010) [17]	Cervical spine fusion	Health Transition Item of the SF-36	Yes	ROC	4.1	-
Merkies, I.S.(2010) [18]	Chronic inflammatory demyelinating polyradiculoneuropathy (CIDP)	Health Transition Item of the SF-36	No	Mean change (between groups)	8.87	4.13
Xiaoxia Luo(2010) [19]	Cardiac insufficiency	①Health Transition Item of the SF-36②Classification of function capacity of the NYHA	No	Mean change (within group)	**HTI: 9.14** **NYHA: 11.22**	**HTI: 9.4** **NYHA: 10.28**
Ingram, M.(2011) [20]	Temporomandibular joint and muscle disorder (TMJMD)	Post-treatment score (cut-off value: 0.5SD)	No	ROC	2.75	1.46
Auffinger, B.M.(2013) [21]	Cervical spondylotic myelopathy	Health Transition Item of the SF-36	No	Mean change (within and between groups)	**Within group:7.76** **Between groups: 3.01**	**Within group:5.56** **Between groups:5.7**3
Lauche, R.(2013) [22]	Chronic nonspecific neck pain	Health Transition Item of the SF-36	No	ROC	-	-
Antonescu, I.(2014) [23]	Postoperative recovery	Health Transition Item of the SF-36	No	Linear regression	-	-
Carlson, M.L.(2015) [24]	Vestibular Schwannoma	Patient’s rating of change(5-point Likert scales)	Yes	Linear regression	7.8	6.7
Zhou, F.(2015) [25]	Cervical spondylotic myelopathy	Health Transition Item of the SF-36(5-point Likert scales)	Yes	Mean change (within and between groups)、ROC	**Within group:5.44** **Between groups:9.62** **ROC4.09**	**Within group: 3.11** **Between groups: 7.14** **ROC:3.91**
Erez, G.(2016) [26]	Conservatively managed stage 5 chronic kidney disease	Karnofsky(KPS scores)	Yes	Mean change (between groups)	5.7	9.2
Park, K.B.(2017) [27]	Failed Back Surgery Syndrome	Patient’s rating of change (5-point Likert scales)	No	ROC	10.23	4
Badhiwala, J.H.(2018) [28]	Degenerative Cervical Myelopathy	Neck Disability Index (NDI)	Yes	ROC	3.93	3.16
Yuksel, S.(2018) [29]	Adult spinal deformity	Patient’s rating of change (15-point Likert scales)	No	Linear regression	7.33	4.37
Brigden, A.(2018) [30]	Chronic Fatigue Syndrome (CFS)/Myalgic encephalomyelitis (ME) in Children and Adolescents	Patient’s rating of change (7-point Likert scales)	No	Mean change (within group)	8.8	-
Kato, S.(2019) [31]	Cervical laminoplasty	Patient’s rating of change or patient satisfaction (7-point Likert scales)	No	ROC	3.9	-
Carton, P(2020) [32]	Arthroscopic Correction of Sports-Related Femoroacetabular Impingement	Patient’s rating of change (5-point Likert scales)	No	Mean change(between groups)、ROC	-	-
Ogura, K.(2020) [33]	Orthopedic oncology	Patient’s rating of change (5-point Likert scales)	Yes	ROC	-	-
Yao, M.(2020) [34]	lumbar disc herniation	Patient’s rating of change (7-point Likert scales)	No	Mean change (between groups)	4.57	3.59
Pintér, D.(2020) [35]	Generalized or segmental dystonia	Patient’s rating of change (7-point Likert scales)	Yes	Mean change、ROC	4.9	6.6
Kawakami, D.(2021) [36]	Post-intensive care syndrome (PICS)	Patient’s rating of change (7-point Likert scales)	No	Mean change (between groups)	6.5	8
Kang, M. (2021) [37]	Idiopathic pulmonary fibrosis	Health Transition Item of the SF-36	Yes	ROC	7	-
Filan, D.(2021) [38]	Femoral acetabular impingement (FAI)	Patient’s rating of change(5-point Likert scales)	Yes	Mean change (between groups)	17.4	3.7
Fu,V.(2021) [39]	Stroke	Health Transition Item of the SF-36	Yes	Linear regression	3	-
Hara, T.(2022)[40]	Gastrointestinal cancer	SF-36 physical functioning subscale scores (cut-off value: the average score of the general Japanese population)	Yes	ROC	-	-

For the selection of statistical methods, the traditional methods were still mainly used, among which the mean change was the most, followed by the ROC curve, and the linear regression method is the least. Most studies calculated the PCS and MCS scores of the SF-36 scale, where the MCID threshold range of the PCS was 2–17.4 and the MCID threshold range of the MCS was 1.46–10.28. And different anchors or statistical methods give different results.

## Discussion

This study systematically summarised and analysed the anchor design based on the existing research of using anchor-based method to ascertain MCID. Although this study did not fully cover all the research, it was representative enough. For the design of anchors, the existing research mainly used subjective anchors. In general, although the types of anchors used in the existing research were different, some rules can still be summarised, which can provide references and suggestions for further research.

For the design of the anchor, firstly, the existing research mainly used subjective anchors. This could be due to the fact that the existing research objects were still mainly PROs, and the disease field involved was mainly functional diseases. Therefore, there are few objective indicators that can reflect changes in diseases, making it difficult to find appropriate objective anchors. However, from the perspective of methodology, the results of MCID estimation using objective anchors are more stable and reliable than those using subjective anchors. This is because subjective anchors are prone to memory bias, which can lead to some bias in the estimated MCID. Secondly, some existing research has used the internal items of the research scale as anchors. For example, when ascertaining the MCID of SF-36, using its internal Health Transition Item or its dimension scores as the anchor, this may be problematic because the “externality” of the anchor-based method is not reflected, and the estimation results may not be reliable enough. In addition, this study found that almost half of the included literature did not test the correlation between anchors and research scales or indicators, which may also lead to unreliable results. In this regard, it is suggested that the priority in selecting anchors should be: objective anchor > anchor with MCID established in subjective anchor (special scale > generic scale) > rank variable anchor in subjective anchor (the cut-off point value of rank anchor is difficult to determine compared to that of MCID established anchor)), and avoid selecting internal anchor. The selected anchor must assess the correlation test (correlation coefficient > 0.3) before selecting the anchor to ascertain the MCID. If it does not reach 0.3, consider using the distribution-based method to ascertain MCID. At the same time, it is recommended that multiple anchors can be selected to ascertain MCID because one of the major shortcomings of the anchor-based method is that the estimated MCID may change with the change of the anchor, and when ascertaining the MCID of different dimensions of a scale, the correlation between the same anchor and different dimensions of the scale may not be the same, and the robustness and complementarity of the results obtained by checking the use of multiple anchors, the MCID can be estimated more accurately [41].

After selecting the appropriate anchor, the patients shall be grouped according to the cut-off values, and then the MCID can only be further calculated using the anchor-based method if the groups are statistically significant and can represent clinically significant changes [42]. A common limitation was the small sample size in the anchor categories. If the number of patients in each anchor category is insufficient, then the resulting MCID may not be reliable and robustness in this case is questionable. The appropriate sample size of each category should be calculated systematically before the investigation, and interim analysis and determination can be carried out during the investigation. If the sample size of the MCID group is small, a larger sample size should be considered [41]. In addition, because there are many types of rank anchors, there is no unified consensus on the cut-off values. The cut-off value and grouping in existing research can be used as a reference (Table 2). It can be adjusted flexibly according to the research design. For example, if the number of people choosing a grade option in the rank anchor is too low, it may be considered to combine it with its neighbouring rank options for the calculation. Moreover, there were two studies in which the MCID of the SF-36 was specifically determined, with the cut-off values based directly on the statistical distribution of their scores. In fact, it can be understood as using the distribution-based method to estimate the MCID of the anchor, but the distribution-based method is not accurate enough to estimate the MCID, so it may be questionable.

Through an in-depth analysis of the statistical process of the MCID of the SF-36 scale, we found that the current selection of statistical methods was still mainly the traditional method, and the results of its MCID threshold values (taking SF-36 PCS and SF-36 MCS as examples) range were relatively large, and the results of MCID were slightly different due to different anchors or statistical methods. Therefore, it is of great significance to select more types of anchors and use more reliable statistical methods to calculate the MCID.

In summary, further research is needed to standardise the anchor design of the anchor-based method, for example by creating a standardised list of anchor choices and priority levels according to different disease types or study indicators, and by specifying the corresponding cut-off values. And more studies should be conducted using the standardised anchor-based method to calculate the MCID. This study also has some limitations. First, all the literature on anchor-based method calculation of MCID are not available. Second, due to the large scope of research, this study did not conduct in-depth mining on the MCID calculation process of scales or clinical indicators in some specific disease areas, which still needs further exploration.

## Conclusion

This study systematically searched the published literature on MCID calculation using the anchor-based method. And the anchor design was summarised and analysed. The results showed that for the design of anchors, subjective anchors were mainly adopted, which were mainly the patient’s rating of change or patient satisfaction and related scale health status evaluation items or scores. Almost half of the studies did not assess the correlation test between the anchor and the research indicator or scale. The cut-off values and grouping were usually based on the selection of the anchor types. Due to the large number of included studies, this review selected the most calculated SF-36 (28 articles) for an in-depth analysis. The results showed that the overall design of the anchor and the cut-off value were the same as above. The statistical methods were mostly the traditional method (mean change、ROC). The MCID threshold values of these studies had a wide range (SF-36 PCS: 2–17.4, SF-36 MCS: 1.46–10.28), and different anchors or statistical methods lead to different results. It is important to select different types of anchors and use more reliable statistical methods to calculate the MCID. The priority of anchor selection is suggested to be objective anchors > anchor with MCID established in subjective anchors (special scale > generic scale) > rank anchor in subjective anchors. Internal anchors should be avoided and anchors should be evaluated by correlation test.

## Supplementary Information

**Additional file 1.**

## Data Availability

The datasets used and/or analysed during the current study are available from the corresponding author on reasonable request.

## Abbreviations

MCID: Minimum Clinically Important Difference
SGRQ: St George's Respiratory Questionnaire
GROC: Global Rating of Change Scale
CGI: Clinical Global Impression
PRO: Patient-reported Outcome
