# Supplementary material for: The anchor design of anchor-based method to determine the minimal clinically important difference: a systematic review
Source: Health Qual Life Outcomes. 2023 Jul 15;21:74. doi: 10.1186/s12955-023-02157-3 (PMC10350268; doi:10.1186/s12955-023-02157-3)
Supplement: Supplementary file 1 — Additional file 1. [file 12955_2023_2157_MOESM1_ESM.docx]

**The anchor design of anchor-based method to determine the** **minimal clinically important difference: a systematic review**

**Appendix 1: A list of 340 literatures**

**Reference^1-249250-340^**

1. Wells G, Li T, Maxwell L, MacLean R, Tugwell P. Determining the minimal clinically important differences in activity, fatigue, and sleep quality in patients with rheumatoid arthritis. *J Rheumatol*. Feb 2007;34(2):280-9.

2. Lee BB, King MT, Simpson JM, et al. Validity, responsiveness, and minimal important difference for the SF-6D health utility scale in a spinal cord injured population. *Value Health*. Jul-Aug 2008;11(4):680-8. doi:10.1111/j.1524-4733.2007.00311.x

3. Lin KC, Hsieh YW, Wu CY, Chen CL, Jang Y, Liu JS. Minimal detectable change and clinically important difference of the Wolf Motor Function Test in stroke patients. *Neurorehabil Neural Repair*. Jun 2009;23(5):429-34. doi:10.1177/1545968308331144

4. Coteur G, Feagan B, Keininger DL, Kosinski M. Evaluation of the meaningfulness of health-related quality of life improvements as assessed by the SF-36 and the EQ-5D VAS in patients with active Crohn's disease. *Aliment Pharmacol Ther*. May 1 2009;29(9):1032-41. doi:10.1111/j.1365-2036.2009.03966.x

5. Purcell A, Fleming J, Bennett S, Burmeister B, Haines T. Determining the minimal clinically important difference criteria for the Multidimensional Fatigue Inventory in a radiotherapy population. *Support Care Cancer*. Mar 2010;18(3):307-15. doi:10.1007/s00520-009-0653-z

6. Lin KC, Fu T, Wu CY, et al. Minimal detectable change and clinically important difference of the Stroke Impact Scale in stroke patients. *Neurorehabil Neural Repair*. Jun 2010;24(5):486-92. doi:10.1177/1545968309356295

7. Merkies IS, van Nes SI, Hanna K, Hughes RA, Deng C. Confirming the efficacy of intravenous immunoglobulin in CIDP through minimum clinically important differences: shifting from statistical significance to clinical relevance. *J Neurol Neurosurg Psychiatry*. Nov 2010;81(11):1194-9. doi:10.1136/jnnp.2009.194324

8. du Bois RM, Weycker D, Albera C, et al. Forced vital capacity in patients with idiopathic pulmonary fibrosis: test properties and minimal clinically important difference. *Am J Respir Crit Care Med*. Dec 15 2011;184(12):1382-9. doi:10.1164/rccm.201105-0840OC

9. Rosen RC, Allen KR, Ni X, Araujo AB. Minimal clinically important differences in the erectile function domain of the International Index of Erectile Function scale. *Eur Urol*. Nov 2011;60(5):1010-6. doi:10.1016/j.eururo.2011.07.053

10. Michener LA, Snyder AR, Leggin BG. Responsiveness of the numeric pain rating scale in patients with shoulder pain and the effect of surgical status. *J Sport Rehabil*. Feb 2011;20(1):115-28. doi:10.1123/jsr.20.1.115

11. de Kleijn WP, De Vries J, Wijnen PA, Drent M. Minimal (clinically) important differences for the Fatigue Assessment Scale in sarcoidosis. *Respir Med*. Sep 2011;105(9):1388-95. doi:10.1016/j.rmed.2011.05.004

12. Maringwa J, Quinten C, King M, et al. Minimal clinically meaningful differences for the EORTC QLQ-C30 and EORTC QLQ-BN20 scales in brain cancer patients. *Ann Oncol*. Sep 2011;22(9):2107-2112. doi:10.1093/annonc/mdq726

13. Zeng L, Chow E, Zhang L, et al. An international prospective study establishing minimal clinically important differences in the EORTC QLQ-BM22 and QLQ-C30 in cancer patients with bone metastases. *Support Care Cancer*. Dec 2012;20(12):3307-13. doi:10.1007/s00520-012-1484-x

14. Araujo AB, Allen KR, Ni X, Rosen RC. Minimal clinically important differences in the vaginal insertion and successful intercourse items of the sexual encounter profile. *J Sex Med*. Jan 2012;9(1):169-79. doi:10.1111/j.1743-6109.2011.02506.x

15. Malay S, Chung KC. The minimal clinically important difference after simple decompression for ulnar neuropathy at the elbow. *J Hand Surg Am*. Apr 2013;38(4):652-9. doi:10.1016/j.jhsa.2013.01.022

16. Auffinger BM, Lall RR, Dahdaleh NS, et al. Measuring surgical outcomes in cervical spondylotic myelopathy patients undergoing anterior cervical discectomy and fusion: assessment of minimum clinically important difference. *PLoS One*. 2013;8(6):e67408. doi:10.1371/journal.pone.0067408

17. Winterstein AP, McGuine TA, Carr KE, Hetzel SJ. Comparison of IKDC and SANE Outcome Measures Following Knee Injury in Active Female Patients. *Sports Health*. Nov 2013;5(6):523-9. doi:10.1177/1941738113499300

18. Lauche R, Langhorst J, Dobos GJ, Cramer H. Clinically meaningful differences in pain, disability and quality of life for chronic nonspecific neck pain - a reanalysis of 4 randomized controlled trials of cupping therapy. *Complement Ther Med*. Aug 2013;21(4):342-7. doi:10.1016/j.ctim.2013.04.005

19. London DA, Stepan JG, Calfee RP. Determining the Michigan Hand Outcomes Questionnaire minimal clinically important difference by means of three methods. *Plast Reconstr Surg*. Mar 2014;133(3):616-625. doi:10.1097/prs.0000000000000034

20. Higaki T, Okano M, Kariya S, et al. Determining minimal clinically important differences in Japanese cedar/cypress pollinosis patients. *Allergol Int*. Dec 2013;62(4):487-93. doi:10.2332/allergolint.13-OA-0570

21. Cheung YT, Foo YL, Shwe M, et al. Minimal clinically important difference (MCID) for the functional assessment of cancer therapy: cognitive function (FACT-Cog) in breast cancer patients. *J Clin Epidemiol*. Jul 2014;67(7):811-20. doi:10.1016/j.jclinepi.2013.12.011

22. Sagberg LM, Jakola AS, Solheim O. Quality of life assessed with EQ-5D in patients undergoing glioma surgery: what is the responsiveness and minimal clinically important difference? *Qual Life Res*. Jun 2014;23(5):1427-34. doi:10.1007/s11136-013-0593-4

23. Katzberg HD, Barnett C, Merkies IS, Bril V. Minimal clinically important difference in myasthenia gravis: outcomes from a randomized trial. *Muscle Nerve*. May 2014;49(5):661-5. doi:10.1002/mus.23988

24. Kon SS, Dilaver D, Mittal M, et al. The Clinical COPD Questionnaire: response to pulmonary rehabilitation and minimal clinically important difference. *Thorax*. Sep 2014;69(9):793-8. doi:10.1136/thoraxjnl-2013-204119

25. Antonescu I, Scott S, Tran TT, Mayo NE, Feldman LS. Measuring postoperative recovery: what are clinically meaningful differences? *Surgery*. Aug 2014;156(2):319-27. doi:10.1016/j.surg.2014.03.005

26. Zhou F, Zhang Y, Sun Y, Zhang F, Pan S, Liu Z. Assessment of the minimum clinically important difference in neurological function and quality of life after surgery in cervical spondylotic myelopathy patients: a prospective cohort study. *Eur Spine J*. Dec 2015;24(12):2918-23. doi:10.1007/s00586-015-4208-3

27. Zanini A, Aiello M, Adamo D, et al. Estimation of minimal clinically important difference in EQ-5D visual analog scale score after pulmonary rehabilitation in subjects with COPD. *Respir Care*. Jan 2015;60(1):88-95. doi:10.4187/respcare.03272

28. Pettersson S, Lundberg IE, Liang MH, Pouchot J, Henriksson EW. Determination of the minimal clinically important difference for seven measures of fatigue in Swedish patients with systemic lupus erythematosus. *Scand J Rheumatol*. May 2015;44(3):206-10. doi:10.3109/03009742.2014.988173

29. Chen P, Lin KC, Liing RJ, Wu CY, Chen CL, Chang KC. Validity, responsiveness, and minimal clinically important difference of EQ-5D-5L in stroke patients undergoing rehabilitation. *Qual Life Res*. Jun 2016;25(6):1585-96. doi:10.1007/s11136-015-1196-z

30. Raman S, Ding K, Chow E, et al. Minimal clinically important differences in the EORTC QLQ-BM22 and EORTC QLQ-C15-PAL modules in patients with bone metastases undergoing palliative radiotherapy. *Qual Life Res*. Oct 2016;25(10):2535-2541. doi:10.1007/s11136-016-1308-4

31. Kaleth AS, Slaven JE, Ang DC. Determining the Minimal Clinically Important Difference for 6-Minute Walk Distance in Fibromyalgia. *Am J Phys Med Rehabil*. Oct 2016;95(10):738-45. doi:10.1097/phm.0000000000000485

32. Zuckerman SL, Chotai S, Devin CJ, et al. Surgical Resection of Intradural Extramedullary Spinal Tumors: Patient Reported Outcomes and Minimum Clinically Important Difference. *Spine (Phila Pa 1976)*. Dec 15 2016;41(24):1925-1932. doi:10.1097/brs.0000000000001653

33. Erez G, Selman L, Murtagh FE. Measuring health-related quality of life in patients with conservatively managed stage 5 chronic kidney disease: limitations of the Medical Outcomes Study Short Form 36: SF-36. *Qual Life Res*. Nov 2016;25(11):2799-2809. doi:10.1007/s11136-016-1313-7

34. Chan A, Yo TE, Wang XJ, et al. Minimal Clinically Important Difference of the Multidimensional Fatigue Symptom Inventory-Short Form (MFSI-SF) for Fatigue Worsening in Asian Breast Cancer Patients. *J Pain Symptom Manage*. Mar 2018;55(3):992-997.e2. doi:10.1016/j.jpainsymman.2017.10.014

35. Lee N, Thompson NR, Passek S, Stilphen M, Katzan IL. Minimally Clinically Important Change in the Activity Measure for Post-Acute Care (AM-PAC), a Generic Patient-Reported Outcome Tool, in People With Low Back Pain. *Phys Ther*. Nov 1 2017;97(11):1094-1102. doi:10.1093/ptj/pzx083

36. Chowdhury NI, Mace JC, Bodner TE, et al. Investigating the minimal clinically important difference for SNOT-22 symptom domains in surgically managed chronic rhinosinusitis. *Int Forum Allergy Rhinol*. Dec 2017;7(12):1149-1155. doi:10.1002/alr.22028

37. Amaral R, Carneiro AC, Wandalsen G, Fonseca JA, Sole D. Control of Allergic Rhinitis and Asthma Test for Children (CARATKids): Validation in Brazil and cutoff values. *Ann Allergy Asthma Immunol*. May 2017;118(5):551-556.e2. doi:10.1016/j.anai.2017.02.007

38. Gervasoni E, Jonsdottir J, Montesano A, Cattaneo D. Minimal Clinically Important Difference of Berg Balance Scale in People With Multiple Sclerosis. *Arch Phys Med Rehabil*. Feb 2017;98(2):337-340.e2. doi:10.1016/j.apmr.2016.09.128

39. Malec JF, Kean J, Monahan PO. The Minimal Clinically Important Difference for the Mayo-Portland Adaptability Inventory. *J Head Trauma Rehabil*. Jul/Aug 2017;32(4):E47-e54. doi:10.1097/htr.0000000000000268

40. Levy JM, Mace JC, Bodner TE, Alt JA, Smith TL. Defining the minimal clinically important difference for olfactory outcomes in the surgical treatment of chronic rhinosinusitis. *Int Forum Allergy Rhinol*. Aug 2017;7(8):821-826. doi:10.1002/alr.21964

41. Smid DE, Franssen FM, Houben-Wilke S, et al. Responsiveness and MCID Estimates for CAT, CCQ, and HADS in Patients With COPD Undergoing Pulmonary Rehabilitation: A Prospective Analysis. *J Am Med Dir Assoc*. Jan 2017;18(1):53-58. doi:10.1016/j.jamda.2016.08.002

42. Kafaja S, Clements PJ, Wilhalme H, et al. Reliability and minimal clinically important differences of forced vital capacity: Results from the Scleroderma Lung Studies (SLS-I and SLS-II). *Am J Respir Crit Care Med*. Mar 1 2018;197(5):644-652. doi:10.1164/rccm.201709-1845OC

43. Raman S, Ding K, Chow E, et al. Minimal clinically important differences in the EORTC QLQ-C30 and brief pain inventory in patients undergoing re-irradiation for painful bone metastases. *Qual Life Res*. Apr 2018;27(4):1089-1098. doi:10.1007/s11136-017-1745-8

44. Gardner AW, Montgomery PS, Wang M. Minimal clinically important differences in treadmill, 6-minute walk, and patient-based outcomes following supervised and home-based exercise in peripheral artery disease. *Vasc Med*. Aug 2018;23(4):349-357. doi:10.1177/1358863x18762599

45. Akaberi A, Klok FA, Cohn DM, Hirsch A, Granton J, Kahn SR. Determining the minimal clinically important difference for the PEmbQoL questionnaire, a measure of pulmonary embolism-specific quality of life. *J Thromb Haemost*. Dec 2018;16(12):2454-2461. doi:10.1111/jth.14302

46. Gravbrot N, Kelly DF, Milligan J, et al. The Minimal Clinically Important Difference of the Anterior Skull Base Nasal Inventory-12. *Neurosurgery*. Aug 1 2018;83(2):277-280. doi:10.1093/neuros/nyx401

47. Ogura K, Yakoub MA, Christ AB, et al. What Are the Minimum Clinically Important Differences in SF-36 Scores in Patients with Orthopaedic Oncologic Conditions? *Clin Orthop Relat Res*. Sep 2020;478(9):2148-2158. doi:10.1097/corr.0000000000001341

48. Quinten C, Kenis C, Decoster L, et al. Determining clinically important differences in health-related quality of life in older patients with cancer undergoing chemotherapy or surgery. *Qual Life Res*. Mar 2019;28(3):663-676. doi:10.1007/s11136-018-2062-6

49. Zanini A, Crisafulli E, D'Andria M, et al. Minimum Clinically Important Difference in 30-s Sit-to-Stand Test After Pulmonary Rehabilitation in Subjects With COPD. *Respir Care*. Oct 2019;64(10):1261-1269. doi:10.4187/respcare.06694

50. Wang D, Chang B, Coxe FR, et al. Clinically Meaningful Improvement After Treatment of Cartilage Defects of the Knee With Osteochondral Grafts. *Am J Sports Med*. Jan 2019;47(1):71-81. doi:10.1177/0363546518808030

51. Lemay KR, Tulloch HE, Pipe AL, Reed JL. Establishing the Minimal Clinically Important Difference for the Hospital Anxiety and Depression Scale in Patients With Cardiovascular Disease. *J Cardiopulm Rehabil Prev*. Nov 2019;39(6):E6-e11. doi:10.1097/hcr.0000000000000379

52. Tillett W, Lin CY, Zbrozek A, Sprabery AT, Birt J. A Threshold of Meaning for Work Disability Improvement in Psoriatic Arthritis Measured by the Work Productivity and Activity Impairment Questionnaire. *Rheumatol Ther*. Sep 2019;6(3):379-391. doi:10.1007/s40744-019-0155-5

53. Nolan CM, Birring SS, Maddocks M, et al. King's Brief Interstitial Lung Disease questionnaire: responsiveness and minimum clinically important difference. *Eur Respir J*. Sep 2019;54(3)doi:10.1183/13993003.00281-2019

54. Siviero P, Limongi F, Gesmundo A, et al. Minimal clinically important decline in physical function over one year: EPOSA study. *BMC Musculoskelet Disord*. May 17 2019;20(1):227. doi:10.1186/s12891-019-2593-1

55. Yeo F, Ng CC, Loh KWJ, et al. Minimal clinically important difference of the EORTC QLQ-CIPN20 for worsening peripheral neuropathy in patients receiving neurotoxic chemotherapy. *Support Care Cancer*. Dec 2019;27(12):4753-4762. doi:10.1007/s00520-019-04771-8

56. Ogura Y, Ogura K, Kobayashi Y, et al. Minimally clinically important differences for the Japanese Orthopaedic Association Back Pain Evaluation Questionnaire (JOABPEQ) following decompression surgery for lumbar spinal stenosis. *J Clin Neurosci*. Nov 2019;69:93-96. doi:10.1016/j.jocn.2019.08.025

57. Agarwalla A, Gowd AK, Liu JN, et al. Predictive Factors and Duration to Return to Sport After Isolated Meniscectomy. *Orthop J Sports Med*. Apr 2019;7(4):2325967119837940. doi:10.1177/2325967119837940

58. Nishitani K, Yamamoto Y, Furu M, et al. The minimum clinically important difference for the Japanese version of the new Knee Society Score (2011KSS) after total knee arthroplasty. *J Orthop Sci*. Nov 2019;24(6):1053-1057. doi:10.1016/j.jos.2019.09.001

59. Iwakura M, Okura K, Kubota M, et al. Estimation of minimal clinically important difference for quadriceps and inspiratory muscle strength in older outpatients with chronic obstructive pulmonary disease: a prospective cohort study. *Phys Ther Res*. 2021;24(1):35-42. doi:10.1298/ptr.E10049

60. Alzyoud J, Medley A, Thompson M, Csiza L. Responsiveness, minimal detectable change, and minimal clinically important difference of the sitting balance scale and function in sitting test in people with stroke. *Physiother Theory Pract*. Feb 2022;38(2):327-336. doi:10.1080/09593985.2020.1756016

61. Ye ZJ, Zhang Z, Tang Y, et al. Minimum clinical important difference for resilience scale specific to cancer: a prospective analysis. *Health Qual Life Outcomes*. Dec 9 2020;18(1):381. doi:10.1186/s12955-020-01631-6

62. Chan ACM, Pang MYC, Ouyang H, Jehu DAM. Minimal Clinically Important Difference of Four Commonly Used Balance Assessment Tools in Individuals after Total Knee Arthroplasty: A Prospective Cohort Study. *Pm r*. Mar 2020;12(3):238-245. doi:10.1002/pmrj.12226

63. Ogura Y, Ogura K, Kobayashi Y, et al. Minimum clinically important difference of major patient-reported outcome measures in patients undergoing decompression surgery for lumbar spinal stenosis. *Clin Neurol Neurosurg*. Sep 2020;196:105966. doi:10.1016/j.clineuro.2020.105966

64. Chen CL, Shen IH, Huang HH, et al. Responsiveness and minimal clinically important difference of TNO-AZL Preschool Children Quality of Life in children with cerebral palsy. *Qual Life Res*. Mar 2020;29(3):825-831. doi:10.1007/s11136-019-02370-y

65. Rahman H, Pipinos, II, Johanning JM, et al. Claudicating patients with peripheral artery disease have meaningful improvement in walking speed after supervised exercise therapy. *J Vasc Surg*. Dec 2021;74(6):1987-1995. doi:10.1016/j.jvs.2021.04.069

66. Takenaka H, Kamiya M, Sugiura H, et al. Responsiveness and Minimal Clinically Important Difference of the 6-minute Walk Distance in Patients Undergoing Lumbar Spinal Canal Stenosis Surgery. *Clin Spine Surg*. Apr 1 2022;35(3):E345-e350. doi:10.1097/bsd.0000000000001196

67. Ibaseta A, Rahman R, Andrade NS, et al. Determining validity, discriminant ability, responsiveness, and minimal clinically important differences for PROMIS in adult spinal deformity. *J Neurosurg Spine*. Feb 19 2021:1-9. doi:10.3171/2020.8.Spine191551

68. Lynch CP, Cha EDK, Mohan S, Geoghegan CE, Jadczak CN, Singh K. Two-year validation and minimal clinically important difference of the Veterans RAND 12 Item Health Survey Physical Component Score in patients undergoing minimally invasive transforaminal lumbar interbody fusion. *J Neurosurg Spine*. Nov 19 2021:1-10. doi:10.3171/2021.6.Spine21231

69. Kang M, Veeraraghavan S, Martin GS, Kempker JA. An updated approach to determine minimal clinically important differences in idiopathic pulmonary fibrosis. *ERJ Open Res*. Oct 2021;7(4)doi:10.1183/23120541.00142-2021

70. Peyton CC, Henriksen C, Reich RR, Azizi M, Gilbert SM. Estimating Minimally Important Differences for the Bladder Cancer Index Using Distribution and Anchor Based Approaches. *J Urol*. Apr 2019;201(4):709-714. doi:10.1016/j.juro.2018.10.008

71. Tremblay G, Daniele P, Breeze J, et al. Quality of life analyses in patients with multiple myeloma: results from the Selinexor (KPT-330) Treatment of Refractory Myeloma (STORM) phase 2b study. *BMC Cancer*. Sep 6 2021;21(1):993. doi:10.1186/s12885-021-08453-9

72. Tamura S, Miyata K, Kobayashi S, Takeda R, Iwamoto H. Minimal clinically important difference of the Berg Balance Scale score in older adults with hip fractures. *Disabil Rehabil*. Aug 19 2021:1-6. doi:10.1080/09638288.2021.1962993

73. Tamura S, Miyata K, Kobayashi S, Takeda R, Iwamoto H. The minimal clinically important difference in Berg Balance Scale scores among patients with early subacute stroke: a multicenter, retrospective, observational study. *Top Stroke Rehabil*. Sep 2022;29(6):423-429. doi:10.1080/10749357.2021.1943800

74. Batting M, Hannink E, Barker K. Minimal clinically important difference of the Four Square Step Test in people with degenerative spinal conditions. *Physiotherapy*. Jun 2022;115:58-60. doi:10.1016/j.physio.2021.04.001

75. Jehu DA, Davis JC, Madden K, Parmar N, Liu-Ambrose T. Minimal Clinically Important Difference of Executive Function Performance in Older Adults Who Fall: A Secondary Analysis of a Randomized Controlled Trial. *Gerontology*. 2022;68(7):771-779. doi:10.1159/000518939

76. Oliveira A, Rebelo P, Paixão C, et al. Minimal Clinically Important Difference for Quadriceps Muscle Strength in People with COPD following Pulmonary Rehabilitation. *Copd*. Feb 2021;18(1):35-44. doi:10.1080/15412555.2021.1874897

77. Duong T, Canbek J, Birkmeier M, et al. The Minimal Clinical Important Difference (MCID) in Annual Rate of Change of Timed Function Tests in Boys with DMD. *J Neuromuscul Dis*. 2021;8(6):939-948. doi:10.3233/jnd-210646

78. Corrini C, Torchio A, Anastasi D, et al. Minimal clinically important difference of modified dynamic gait index in people with neurological disorders. *Gait Posture*. Oct 2021;90:210-214. doi:10.1016/j.gaitpost.2021.08.024

79. Fu V, Weatherall M, McNaughton H. Estimating the minimal clinically important difference for the Physical Component Summary of the Short Form 36 for patients with stroke. *J Int Med Res*. Dec 2021;49(12):3000605211067902. doi:10.1177/03000605211067902

80. Passias PG, Pierce KE, Williamson T, et al. Establishing the minimal clinically important difference for the PROMIS Physical domains in cervical deformity patients. *J Clin Neurosci*. Feb 2022;96:19-24. doi:10.1016/j.jocn.2021.12.008

81. Hara T, Kogure E, Iijima S, Fukawa Y, Kubo A, Kakuda W. Minimal clinically important difference in postoperative recovery among patients with gastrointestinal cancer. *Support Care Cancer*. Mar 2022;30(3):2197-2205. doi:10.1007/s00520-021-06632-9

82. Wellons RD, Duhe SE, MacDowell SG, Hodge A, Oxborough S, Levitzky EE. Estimating the minimal clinically important difference for balance and gait outcome measures in individuals with vestibular disorders. *J Vestib Res*. 2022;32(3):223-233. doi:10.3233/ves-201630

83. HUANG Xin-ping, WENG Min, WAN Chong-hua, YANG Zheng, XU Chuan-zhi, ZHANG Xiao-pan. Study on Minimal Important Difference of The Quality of Life Instrument for Patients with Chronic Renal Failure (QLICD-CRF). 2012:

84. LI Wei. Development and Preliminary Applications of the Quality of Life Instrument for Cancer Patients-Liver Cancer. Kunming Medical University; 2013.

85. YANG Zheng. Development of the General Module of the system of Quality of Life Instuments for Cancer Patients(V2．0)and Estimation of its Minimal Clinically Important Difference. Southern Medical University; 2015.

86. YU Lei. Development and Validation of Quality of Life Instrument for Patients with Chronic Gastritis(QLICD—CG V2．0)and Peptic Ulcer(QLICD—PU V2．0)and Estimations of Their Minimal Clinically Important Difference. Guangdong Medical College; 2015.

87. YAO Danni. The development and evaluation of “Main Symptom scale for Psoriasis Vulgaris"and Minimal Clinically Important Differences of the Dermatology Life Quality Index. Guangzhou University of Chinese Medicine; 2015.

88. XUAN Hui. *Development of the Colorectal Cancer Scale (V2.0) among the System of Quality of Life Instruments for Cancer Patients and Estimations of its Minimal Clinically Important Differences*. Guangdong Medical College; 2016.

89. XI Junding. Development and Validation of the Quality of Life Instruments for Patients with schizophrenia and anxiety disorder and Establishment of their Minimal Clinically Important Differences. Guangdong Medical College; 2016.

90. ZHAO Mengdi, Xi Junding, WAN Chonghua, et al. Establishing minimal clinically importance difference value of life quality determination of the scale for patient with schizophrenia. *Soft Science of Health.* 2019;33(8):4.

91. SUN Hui, XIE Yang, LIJiansheng. Preliminary determination and verification of minimum clinically important difference of COPD-PRO scale based on anchor-based method combined with distribution-based method. 2016:

92. CHEN Mingyang. Development and Validation of the Patient Report Outcomes Instruments for Patients with rheumatoid arthritis and systemic lupus erythematosus and Establishment of their Minimal Clinically Important Differences. Guangdong Medical University; 2017.

93. ZHANG Chuanmeng. Development of the Patient Reported Outcome for Coronary Heart Disease and Estimation of its Minimal Clinically Important Difference. Guangdong Medical University; 2017.

94. LUO Jian. The Research of Stroke Patients Quality of Life and Formulate Its Minimal Clinically Important Difference. Kunming Medical University; 2017.

95. LIU Xu. Development and Validation of the Quality of Life Instrument for Patients with Gout (V2.0) and its Formulate method of Clinical Minimal Important Different. Guangdong Medical University; 2017.

96. ZHOU Lin. Development of Quality of Life Instrument for Cervical Cancer Patients (QLICP-CE V2.0) and Estimation of its Minimal Clinically Important Difference. Guangdong Medical University; 2018.

97. RUAN Jinhao. Development and Validation of the Patient Report Outcomes Instruments for Patients with Chronic obstructive pulmonary disease and Establishment of its Minimal Clinically Important Difference. Guangdong Medical University; 2018.

98. SHEN Fangli. Development of the Quality of Life Instrument for Hyperthyroidism and Estimation of its Minimal Clinically Important Difference. Guangdong Medical University; 2019.

99. LI Xiaojing. QOL study and MCID Establishment in COPD Patient. Kunming Medical University; 2019.

100. ZHOU Jiali, ZHANG Qingqing, YANG Zhen, et al. Formulation of minimum clinical important difference of diabetic based on QLICD-DM(V2.0) scale. *Soft Science of Health*. 2020;34(5):4.

101. Ren Dandan,Wan Chonghua,Yang Zheng, et al. Study on Anchor-Based Methods to Calculate Minimal Clinically Important Differences and Its Applications in Gastric Cancer. *Chinese Journal of Health Statistics*. 2020;37(4):5.

102. FANG Yangchen，YANG Zheng，WAN Chonghua，WU Jiayuan，HU Liren，LIANG Qilian. Determination of MCID in each field of QLICP-NA. *Shandong Medical Journal*. 2021;61(18):4.

103. LI Fei, LIU Yuxi, WAN Chonghua, et al. Establishing and evaluating of minimum clinical importance differences for the  quality of life instrument in patients with head and neck cancer based on anchor method and distribution method.*Chin J Cancer Prev Treat.* 2021;28(20):1568-1572.

104. Carreon LY, Glassman SD, Campbell MJ, Anderson PA. Neck Disability Index, short form-36 physical component summary, and pain scales for neck and arm pain: the minimum clinically important difference and substantial clinical benefit after cervical spine fusion. *Spine J*. Jun 2010;10(6):469-74. doi:10.1016/j.spinee.2010.02.007

105. TANG Shunding. Research of Patient Reported Outcomes for Hypertension Patients and MCID Development. Kunming Medical University; 2021.

106. Angst F, Aeschlimann A, Stucki G. Smallest detectable and minimal clinically important differences of rehabilitation intervention with their implications for required sample sizes using WOMAC and SF-36 quality of life measurement instruments in patients with osteoarthritis of the lower extremities. *Arthritis Rheum*. Aug 2001;45(4):384-91. doi:10.1002/1529-0131(200108)45:4<384::Aid-art352>3.0.Co;2-0

107. Escobar A, Quintana JM, Bilbao A, Aróstegui I, Lafuente I, Vidaurreta I. Responsiveness and clinically important differences for the WOMAC and SF-36 after total knee replacement. *Osteoarthritis Cartilage*. Mar 2007;15(3):273-80. doi:10.1016/j.joca.2006.09.001

108. Kocks JW, Tuinenga MG, Uil SM, van den Berg JW, Ståhl E, van der Molen T. Health status measurement in COPD: the minimal clinically important difference of the clinical COPD questionnaire. *Respir Res*. Apr 7 2006;7(1):62. doi:10.1186/1465-9921-7-62

109. Hsieh YW, Wang CH, Wu SC, Chen PC, Sheu CF, Hsieh CL. Establishing the minimal clinically important difference of the Barthel Index in stroke patients. *Neurorehabil Neural Repair*. May-Jun 2007;21(3):233-8. doi:10.1177/1545968306294729

110. Lang CE, Edwards DF, Birkenmeier RL, Dromerick AW. Estimating minimal clinically important differences of upper-extremity measures early after stroke. *Arch Phys Med Rehabil*. Sep 2008;89(9):1693-700. doi:10.1016/j.apmr.2008.02.022

111. Dawson J, Doll H, Boller I, et al. Comparative responsiveness and minimal change for the Oxford Elbow Score following surgery. *Qual Life Res*. Dec 2008;17(10):1257-67. doi:10.1007/s11136-008-9409-3

112. Bilbao A, Quintana JM, Escobar A, et al. Responsiveness and clinically important differences for the VF-14 index, SF-36, and visual acuity in patients undergoing cataract surgery. *Ophthalmology*. Mar 2009;116(3):418-424.e1. doi:10.1016/j.ophtha.2008.11.020

113. Shi HY, Lee KT, Lee HH, et al. The minimal clinically important difference in the Gastrointestinal Quality-of-Life Index after cholecystectomy. *Surg Endosc*. Dec 2009;23(12):2708-12. doi:10.1007/s00464-009-0475-6

114. Mannion AF, Porchet F, Kleinstück FS, et al. The quality of spine surgery from the patient's perspective: part 2. Minimal clinically important difference for improvement and deterioration as measured with the Core Outcome Measures Index. *Eur Spine J*. Aug 2009;18 Suppl 3(Suppl 3):374-9. doi:10.1007/s00586-009-0931-y

115. Barnes ML, Vaidyanathan S, Williamson PA, Lipworth BJ. The minimal clinically important difference in allergic rhinitis. *Clin Exp Allergy*. Feb 2010;40(2):242-50. doi:10.1111/j.1365-2222.2009.03381.x

116. Quittner AL, Modi AC, Wainwright C, Otto K, Kirihara J, Montgomery AB. Determination of the minimal clinically important difference scores for the Cystic Fibrosis Questionnaire-Revised respiratory symptom scale in two populations of patients with cystic fibrosis and chronic Pseudomonas aeruginosa airway infection. *Chest*. Jun 2009;135(6):1610-1618. doi:10.1378/chest.08-1190

117. Tashjian RZ, Deloach J, Green A, Porucznik CA, Powell AP. Minimal clinically important differences in ASES and simple shoulder test scores after nonoperative treatment of rotator cuff disease. *J Bone Joint Surg Am*. Feb 2010;92(2):296-303. doi:10.2106/jbjs.H.01296

118. Gerlinger C, Schumacher U, Faustmann T, Colligs A, Schmitz H, Seitz C. Defining a minimal clinically important difference for endometriosis-associated pelvic pain measured on a visual analog scale: analyses of two placebo-controlled, randomized trials. *Health Qual Life Outcomes*. Nov 24 2010;8:138. doi:10.1186/1477-7525-8-138

119. Maughan EF, Lewis JS. Outcome measures in chronic low back pain. *Eur Spine J*. Sep 2010;19(9):1484-94. doi:10.1007/s00586-010-1353-6

120. Kvam AK, Fayers P, Wisloff F. What changes in health-related quality of life matter to multiple myeloma patients? A prospective study. *Eur J Haematol*. Apr 2010;84(4):345-53. doi:10.1111/j.1600-0609.2009.01404.x

121. Wang YC, Hart DL, Stratford PW, Mioduski JE. Baseline dependency of minimal clinically important improvement. *Phys Ther*. May 2011;91(5):675-88. doi:10.2522/ptj.20100229

122. Gremeaux V, Troisgros O, Benaïm S, et al. Determining the minimal clinically important difference for the six-minute walk test and the 200-meter fast-walk test during cardiac rehabilitation program in coronary artery disease patients after acute coronary syndrome. *Arch Phys Med Rehabil*. Apr 2011;92(4):611-9. doi:10.1016/j.apmr.2010.11.023

123. Ingram M, Choi YH, Chiu CY, et al. USE OF THE MINIMAL CLINICALLY IMPORTANT DIFFERENCE (MCID) FOR EVALUATING TREATMENT OUTCOMES WITH TMJMD PATIENTS: A PRELIMINARY STUDY(). *J Appl Biobehav Res*. Dec 1 2011;16(3-4):148-166. doi:10.1111/j.1751-9861.2011.00068.x

124. Adamchic I, Tass PA, Langguth B, et al. Linking the Tinnitus Questionnaire and the subjective Clinical Global Impression: which differences are clinically important? *Health Qual Life Outcomes*. Jul 10 2012;10:79. doi:10.1186/1477-7525-10-79

125. SHEN Xianshan. Establishing the Minimal Clinically Important Difference and Detectable Change of the Stroke Rehabilitation Assessment of Movement. Anhui Medical University; 2012.

126. Bedard G, Zeng L, Zhang L, et al. Minimal clinically important differences in the Edmonton symptom assessment system in patients with advanced cancer. *J Pain Symptom Manage*. Aug 2013;46(2):192-200. doi:10.1016/j.jpainsymman.2012.07.022

127. Kukkonen J, Kauko T, Vahlberg T, Joukainen A, Aärimaa V. Investigating minimal clinically important difference for Constant score in patients undergoing rotator cuff surgery. *J Shoulder Elbow Surg*. Dec 2013;22(12):1650-5. doi:10.1016/j.jse.2013.05.002

128. Clement ND, MacDonald D, Simpson AH. The minimal clinically important difference in the Oxford knee score and Short Form 12 score after total knee arthroplasty. *Knee Surg Sports Traumatol Arthrosc*. Aug 2014;22(8):1933-9. doi:10.1007/s00167-013-2776-5

129. Kim JK, Park ES. Comparative responsiveness and minimal clinically important differences for idiopathic ulnar impaction syndrome. *Clin Orthop Relat Res*. May 2013;471(5):1406-11. doi:10.1007/s11999-013-2843-8

130. McCallum RW, Lembo A, Esfandyari T, et al. Phase 2b, randomized, double-blind 12-week studies of TZP-102, a ghrelin receptor agonist for diabetic gastroparesis. *Neurogastroenterol Motil*. Nov 2013;25(11):e705-17. doi:10.1111/nmo.12184

131. Kwok BC, Pua YH, Mamun K, Wong WP. The minimal clinically important difference of six-minute walk in Asian older adults. *BMC Geriatr*. Mar 6 2013;13:23. doi:10.1186/1471-2318-13-23

132. Hong F, Bosco JL, Bush N, Berry DL. Patient self-appraisal of change and minimal clinically important difference on the European organization for the research and treatment of cancer quality of life questionnaire core 30 before and during cancer therapy. *BMC Cancer*. Mar 28 2013;13:165. doi:10.1186/1471-2407-13-165

133. Den Oudsten BL, Zijlstra WP, De Vries J. The minimal clinical important difference in the World Health Organization Quality of Life instrument--100. *Support Care Cancer*. May 2013;21(5):1295-301. doi:10.1007/s00520-012-1664-8

134. Parker SL, Godil SS, Shau DN, Mendenhall SK, McGirt MJ. Assessment of the minimum clinically important difference in pain, disability, and quality of life after anterior cervical discectomy and fusion: clinical article. *J Neurosurg Spine*. Feb 2013;18(2):154-60. doi:10.3171/2012.10.Spine12312

135. CHE Xiaolu. The study of responsiveness and minimal clinical important difference of the Chronic Gastritis in gastrointestinal disease patient reported outcomes questionnaire. Guangzhou University of Chinese Medicine; 2013.

136. Franchignoni F, Vercelli S, Giordano A, Sartorio F, Bravini E, Ferriero G. Minimal clinically important difference of the disabilities of the arm, shoulder and hand outcome measure (DASH) and its shortened version (QuickDASH). *J Orthop Sports Phys Ther*. Jan 2014;44(1):30-9. doi:10.2519/jospt.2014.4893

137. Ofenloch RF, Diepgen TL, Popielnicki A, et al. Severity and functional disability of patients with occupational contact dermatitis: validation of the German version of the Occupational Contact Dermatitis Disease Severity Index. *Contact Dermatitis*. Feb 2015;72(2):84-9. doi:10.1111/cod.12302

138. van de Water AT, Shields N, Davidson M, Evans M, Taylor NF. Reliability and validity of shoulder function outcome measures in people with a proximal humeral fracture. *Disabil Rehabil*. 2014;36(13):1072-9. doi:10.3109/09638288.2013.829529

139. Beninato M, Fernandes A, Plummer LS. Minimal clinically important difference of the functional gait assessment in older adults. *Phys Ther*. Nov 2014;94(11):1594-603. doi:10.2522/ptj.20130596

140. Paulsen A, Roos EM, Pedersen AB, Overgaard S. Minimal clinically important improvement (MCII) and patient-acceptable symptom state (PASS) in total hip arthroplasty (THA) patients 1 year postoperatively. *Acta Orthop*. Feb 2014;85(1):39-48. doi:10.3109/17453674.2013.867782

141. YANG Yuanyuan. *Development of patient-reported outcomes instrument for cirrhosis of the liver and determine the minimal clinical important difference*. Shanxi Medical University; 2014.

142. Chien A, Lai DM, Cheng CH, Wang SF, Hsu WL, Wang JL. Responsiveness of the Chinese versions of the Japanese Orthopaedic Association Cervical Myelopathy Evaluation Questionnaire and Neck Disability Index in postoperative patients with cervical spondylotic myelopathy. *Spine (Phila Pa 1976)*. Sep 1 2015;40(17):1315-21. doi:10.1097/brs.0000000000001005

143. Torrens C, Guirro P, Santana F. The minimal clinically important difference for function and strength in patients undergoing reverse shoulder arthroplasty. *J Shoulder Elbow Surg*. Feb 2016;25(2):262-8. doi:10.1016/j.jse.2015.07.020

144. Horváth K, Aschermann Z, Ács P, et al. Minimal clinically important difference on the Motor Examination part of MDS-UPDRS. *Parkinsonism Relat Disord*. Dec 2015;21(12):1421-6. doi:10.1016/j.parkreldis.2015.10.006

145. Tanaka Y, Brod M, Lane JR, Upadhyaya H. What Is a Clinically Relevant Improvement in Quality of Life in Adults With ADHD? *J Atten Disord*. Jan 2019;23(1):65-75. doi:10.1177/1087054715580395

146. Mao HF, Kuo CA, Huang WN, Cummings JL, Hwang TJ. Values of the Minimal Clinically Important Difference for the Neuropsychiatric Inventory Questionnaire in Individuals with Dementia. *J Am Geriatr Soc*. Jul 2015;63(7):1448-52. doi:10.1111/jgs.13473

147. Bennett RM, Bushmakin AG, Cappelleri JC, Zlateva G, Sadosky AB. Minimal clinically important difference in the fibromyalgia impact questionnaire. *J Rheumatol*. Jun 2009;36(6):1304-11. doi:10.3899/jrheum.081090

148. Horváth K, Aschermann Z, Ács P, et al. Minimal Clinically Important Difference on Parkinson's Disease Sleep Scale 2nd Version. *Parkinsons Dis*. 2015;2015:970534. doi:10.1155/2015/970534

149. Pereira M, Cruz EB, Domingues L, Duarte S, Carnide F, Fernandes R. Responsiveness and Interpretability of the Portuguese Version of the Neck Disability Index in Patients With Chronic Neck Pain Undergoing Physiotherapy. *Spine (Phila Pa 1976)*. Nov 2015;40(22):E1180-6. doi:10.1097/brs.0000000000001034

150. CHEN Ruiquan, WU Jianxian, SHEN Xianshan. A research on the minimal clinically important differences of chinese version of the Fugl-Meyer motor scale. *Acta Universitatis Medicinalis Anhui*. 2015;50(4):4.

151. Smith-Forbes EV, Howell DM, Willoughby J, Pitts DG, Uhl TL. Specificity of the minimal clinically important difference of the quick Disabilities of the Arm Shoulder and Hand (QDASH) for distal upper extremity conditions. *J Hand Ther*. Jan-Mar 2016;29(1):81-8; quiz 88. doi:10.1016/j.jht.2015.09.003

152. Alma H, de Jong C, Jelusic D, et al. Health status instruments for patients with COPD in pulmonary rehabilitation: defining a minimal clinically important difference. *NPJ Prim Care Respir Med*. Sep 1 2016;26:16041. doi:10.1038/npjpcrm.2016.41

153. Haase I, Winkeler M, Imgart H. [Anchor-based ascertaining of meaningful changes in depressive symptoms using the example of the German short form of the CES-D]. *Neuropsychiatr*. Jun 2016;30(2):82-91. Ankerbasierte Ermittlung klinisch relevanter Veränderung depressiver Symptomatik am Beispiel der Kurzform der CES-D. doi:10.1007/s40211-016-0184-z

154. Beauchamp MK, Harrison SL, Goldstein RS, Brooks D. Interpretability of Change Scores in Measures of Balance in People With COPD. *Chest*. Mar 2016;149(3):696-703. doi:10.1378/chest.15-0717

155. Karagiannopoulos C, Sitler M, Michlovitz S, Tucker C, Tierney R. Responsiveness of the active wrist joint position sense test after distal radius fracture intervention. *J Hand Ther*. Oct-Dec 2016;29(4):474-482. doi:10.1016/j.jht.2016.06.009

156. Nwachukwu BU, Fields K, Chang B, Nawabi DH, Kelly BT, Ranawat AS. Preoperative Outcome Scores Are Predictive of Achieving the Minimal Clinically Important Difference After Arthroscopic Treatment of Femoroacetabular Impingement. *Am J Sports Med*. Mar 2017;45(3):612-619. doi:10.1177/0363546516669325

157. Gaunt DM, Metcalfe C, Ridd M. The Patient-Oriented Eczema Measure in young children: responsiveness and minimal clinically important difference. *Allergy*. Nov 2016;71(11):1620-1625. doi:10.1111/all.12942

158. Fulk GD, Ludwig M, Dunning K, Golden S, Boyne P, West T. How much change in the stroke impact scale-16 is important to people who have experienced a stroke? *Top Stroke Rehabil*. Nov-Dec 2010;17(6):477-83. doi:10.1310/tsr1706-477

159. Myles PS, Myles DB, Galagher W, Chew C, MacDonald N, Dennis A. Minimal Clinically Important Difference for Three Quality of Recovery Scales. *Anesthesiology*. Jul 2016;125(1):39-45. doi:10.1097/aln.0000000000001158

160. Hui D, Shamieh O, Paiva CE, et al. Minimal Clinically Important Difference in the Physical, Emotional, and Total Symptom Distress Scores of the Edmonton Symptom Assessment System. *J Pain Symptom Manage*. Feb 2016;51(2):262-9. doi:10.1016/j.jpainsymman.2015.10.004

161. Werner BC, Chang B, Nguyen JT, Dines DM, Gulotta LV. What Change in American Shoulder and Elbow Surgeons Score Represents a Clinically Important Change After Shoulder Arthroplasty? *Clin Orthop Relat Res*. Dec 2016;474(12):2672-2681. doi:10.1007/s11999-016-4968-z

162. Weller K, Magerl M, Peveling-Oberhag A, Martus P, Staubach P, Maurer M. The Angioedema Quality of Life Questionnaire (AE-QoL) - assessment of sensitivity to change and minimal clinically important difference. *Allergy*. Aug 2016;71(8):1203-9. doi:10.1111/all.12900

163. Nolan CM, Longworth L, Lord J, et al. The EQ-5D-5L health status questionnaire in COPD: validity, responsiveness and minimum important difference. *Thorax*. Jun 2016;71(6):493-500. doi:10.1136/thoraxjnl-2015-207782

164. Falissard B, Sapin C, Loze JY, Landsberg W, Hansen K. Defining the minimal clinically important difference (MCID) of the Heinrichs-carpenter quality of life scale (QLS). *Int J Methods Psychiatr Res*. Jun 2016;25(2):101-11. doi:10.1002/mpr.1483

165. Vishwanathan K, Alizadehkhaiyat O, Kemp GJ, Frostick SP. Minimal clinically important difference of Liverpool Elbow Score in elbow arthroplasty. *JSES Open Access*. Oct 2017;1(3):144-148. doi:10.1016/j.jses.2017.07.004

166. Tashjian RZ, Hung M, Keener JD, et al. Determining the minimal clinically important difference for the American Shoulder and Elbow Surgeons score, Simple Shoulder Test, and visual analog scale (VAS) measuring pain after shoulder arthroplasty. *J Shoulder Elbow Surg*. Jan 2017;26(1):144-148. doi:10.1016/j.jse.2016.06.007

167. Gagnier JJ, Robbins C, Bedi A, Carpenter JE, Miller BS. Establishing minimally important differences for the American Shoulder and Elbow Surgeons score and the Western Ontario Rotator Cuff Index in patients with full-thickness rotator cuff tears. *J Shoulder Elbow Surg*. May 2018;27(5):e160-e166. doi:10.1016/j.jse.2017.10.042

168. Kluger BM, Garimella S, Garvan C. Minimal clinically important difference of the Modified Fatigue Impact Scale in Parkinson's disease. *Parkinsonism Relat Disord*. Oct 2017;43:101-104. doi:10.1016/j.parkreldis.2017.07.016

169. Pang PS, Lane KA, Tavares M, et al. Is there a clinically meaningful difference in patient reported dyspnea in acute heart failure? An analysis from URGENT Dyspnea. *Heart Lung*. Jul-Aug 2017;46(4):300-307. doi:10.1016/j.hrtlng.2017.03.003

170. Park KB, Shin JS, Lee J, et al. Minimum Clinically Important Difference and Substantial Clinical Benefit in Pain, Functional, and Quality of Life Scales in Failed Back Surgery Syndrome Patients. *Spine (Phila Pa 1976)*. Apr 15 2017;42(8):E474-e481. doi:10.1097/brs.0000000000001950

171. Pornsuriyasak P, Thungtitigul P, Kawamatawong T, Birring SS, Pongmesa T. Minimal Clinically Important Differences (MCIDs) of the Thai Version of the Leicester Cough Questionnaire for Subacute and Chronic Cough. *Value Health Reg Issues*. May 2017;12:57-62. doi:10.1016/j.vhri.2017.03.009

172. Myles PS, Myles DB, Galagher W, et al. Measuring acute postoperative pain using the visual analog scale: the minimal clinically important difference and patient acceptable symptom state. *Br J Anaesth*. Mar 1 2017;118(3):424-429. doi:10.1093/bja/aew466

173. Hwang CJ, Ellis R, Davis RM, Tolleson-Rinehart S. Determination of the Minimal Clinically Important Difference of the University of North Carolina Dry Eye Management Scale. *Cornea*. Sep 2017;36(9):1054-1060. doi:10.1097/ico.0000000000001287

174. Naylor JM, Mills K, Buhagiar M, Fortunato R, Wright R. Minimal important improvement thresholds for the six-minute walk test in a knee arthroplasty cohort: triangulation of anchor- and distribution-based methods. *BMC Musculoskelet Disord*. Sep 13 2016;17(1):390. doi:10.1186/s12891-016-1249-7

175. Chan HY, Chen JY, Zainul-Abidin S, Ying H, Koo K, Rikhraj IS. Minimal Clinically Important Differences for American Orthopaedic Foot & Ankle Society Score in Hallux Valgus Surgery. *Foot Ankle Int*. May 2017;38(5):551-557. doi:10.1177/1071100716688724

176. Martín-Fernández J, Gray-Laymón P, Molina-Siguero A, et al. Cross-cultural adaptation and validation of the Spanish version of the Oxford Hip Score in patients with hip osteoarthritis. *BMC Musculoskelet Disord*. May 22 2017;18(1):205. doi:10.1186/s12891-017-1568-3

177. Park I, Lee JH, Hyun HS, Lee TK, Shin SJ. Minimal clinically important differences in Rowe and Western Ontario Shoulder Instability Index scores after arthroscopic repair of anterior shoulder instability. *J Shoulder Elbow Surg*. Apr 2018;27(4):579-584. doi:10.1016/j.jse.2017.10.032

178. Corallo V, Torre M, Ferrara G, et al. What do spinal cord injury patients think of their improvement? A study of the minimal clinically important difference of the Spinal Cord Independence Measure III. *Eur J Phys Rehabil Med*. Aug 2017;53(4):508-515. doi:10.23736/s1973-9087.17.04240-x

179. Monticone M, Ambrosini E, Rocca B, Foti C, Ferrante S. Responsiveness and minimal clinically important changes for the Tampa Scale of Kinesiophobia after lumbar fusion during cognitive behavioral rehabilitation. *Eur J Phys Rehabil Med*. Jun 2017;53(3):351-358. doi:10.23736/s1973-9087.16.04362-8

180. Díaz-Arribas MJ, Fernández-Serrano M, Royuela A, et al. Minimal Clinically Important Difference in Quality of Life for Patients With Low Back Pain. *Spine (Phila Pa 1976)*. Dec 15 2017;42(24):1908-1916. doi:10.1097/brs.0000000000002298

181. Young VN, Jeong K, Rothenberger SD, et al. Minimal clinically important difference of voice handicap index-10 in vocal fold paralysis. *Laryngoscope*. Jun 2018;128(6):1419-1424. doi:10.1002/lary.27001

182. Alma HJ, de Jong C, Jelusic D, et al. Assessing health status over time: impact of recall period and anchor question on the minimal clinically important difference of copd health status tools. *Health Qual Life Outcomes*. Jun 26 2018;16(1):130. doi:10.1186/s12955-018-0950-7

183. Bilbao A, García-Pérez L, Arenaza JC, et al. Psychometric properties of the EQ-5D-5L in patients with hip or knee osteoarthritis: reliability, validity and responsiveness. *Qual Life Res*. Nov 2018;27(11):2897-2908. doi:10.1007/s11136-018-1929-x

184. Hoehle LP, Phillips KM, Speth MM, Caradonna DS, Gray ST, Sedaghat AR. Responsiveness and minimal clinically important difference for the EQ-5D in chronic rhinosinusitis. *Rhinology*. Apr 1 2019;57(2):110-116. doi:10.4193/Rhin18.122

185. Puzzitiello RN, Gowd AK, Liu JN, Agarwalla A, Verma NN, Forsythe B. Establishing minimal clinically important difference, substantial clinical benefit, and patient acceptable symptomatic state after biceps tenodesis. *J Shoulder Elbow Surg*. Apr 2019;28(4):639-647. doi:10.1016/j.jse.2018.09.025

186. Hall DA, Mehta RL, Argstatter H. Interpreting the Tinnitus Questionnaire (German version): what individual differences are clinically important? *Int J Audiol*. Jul 2018;57(7):553-557. doi:10.1080/14992027.2018.1442591

187. Lyman S, Lee YY, McLawhorn AS, Islam W, MacLean CH. What Are the Minimal and Substantial Improvements in the HOOS and KOOS and JR Versions After Total Joint Replacement? *Clin Orthop Relat Res*. Dec 2018;476(12):2432-2441. doi:10.1097/corr.0000000000000456

188. Hui D, Hess K, Dibaj SS, et al. The minimal clinically important difference of the Richmond Agitation-Sedation Scale in patients with cancer with agitated delirium. *Cancer*. May 15 2018;124(10):2246-2252. doi:10.1002/cncr.31312

189. Koorevaar RCT, Kleinlugtenbelt YV, Landman EBM, van 't Riet E, Bulstra SK. Psychological symptoms and the MCID of the DASH score in shoulder surgery. *J Orthop Surg Res*. Oct 4 2018;13(1):246. doi:10.1186/s13018-018-0949-0

190. Benhissen Z, Konzelmann M, Vuistiner P, Léger B, Luthi F, Benaim C. Determining the minimal clinically important difference of the hand function sort questionnaire in vocational rehabilitation. *Ann Phys Rehabil Med*. May 2019;62(3):155-160. doi:10.1016/j.rehab.2018.11.003

191. Stein T, Müller D, Blank M, et al. Stabilization of Acute High-Grade Acromioclavicular Joint Separation: A Prospective Assessment of the Clavicular Hook Plate Versus the Double Double-Button Suture Procedure. *Am J Sports Med*. Sep 2018;46(11):2725-2734. doi:10.1177/0363546518788355

192. Stefanovics EA, Rosenheck RA, Jones KM, Huang G, Krystal JH. Minimal Clinically Important Differences (MCID) in Assessing Outcomes of Post-Traumatic Stress Disorder. *Psychiatr Q*. Mar 2018;89(1):141-155. doi:10.1007/s11126-017-9522-y

193. Simovitch R, Flurin PH, Wright T, Zuckerman JD, Roche CP. Quantifying success after total shoulder arthroplasty: the minimal clinically important difference. *J Shoulder Elbow Surg*. Feb 2018;27(2):298-305. doi:10.1016/j.jse.2017.09.013

194. Agarwalla A, Gowd AK, Liu JN, et al. Predictive Factors and the Duration to Pre-Injury Work Status Following Biceps Tenodesis. *Arthroscopy*. Apr 2019;35(4):1026-1033. doi:10.1016/j.arthro.2018.10.144

195. Clement ND, Bardgett M, Weir D, Holland J, Gerrand C, Deehan DJ. What is the Minimum Clinically Important Difference for the WOMAC Index After TKA? *Clin Orthop Relat Res*. Oct 2018;476(10):2005-2014. doi:10.1097/corr.0000000000000444

196. Hung M, Saltzman CL, Kendall R, et al. What Are the MCIDs for PROMIS, NDI, and ODI Instruments Among Patients With Spinal Conditions? *Clin Orthop Relat Res*. Oct 2018;476(10):2027-2036. doi:10.1097/corr.0000000000000419

197. Braun C, Handoll HH. Estimating the Minimal Important Difference for the Western Ontario Rotator Cuff Index (WORC) in adults with shoulder pain associated with partial-thickness rotator cuff tears. *Musculoskelet Sci Pract*. Jun 2018;35:30-33. doi:10.1016/j.msksp.2018.02.003

198. Beauchamp MK, Ward RE, Jette AM, Bean JF. Meaningful Change Estimates for the Late-Life Function and Disability Instrument in Older Adults. *J Gerontol A Biol Sci Med Sci*. Mar 14 2019;74(4):556-559. doi:10.1093/gerona/gly230

199. Song MJ, Lee JH, Shin WS. Minimal Clinically Important Difference of Berg Balance Scale scores in people with acute stroke. *Physical Therapy Rehabilitation Science*. 2018;7(3):102-108.

200. Espay AJ, Trosch R, Suarez G, Johnson J, Marchese D, Comella C. Minimal clinically important change in the Toronto Western Spasmodic Torticollis Rating Scale. *Parkinsonism Relat Disord*. Jul 2018;52:94-97. doi:10.1016/j.parkreldis.2018.03.002

201. Phillips KM, Hoehle LP, Caradonna DS, Gray ST, Sedaghat AR. Minimal clinically important difference for the 22-item Sinonasal Outcome Test in medically managed patients with chronic rhinosinusitis. *Clin Otolaryngol*. Oct 2018;43(5):1328-1334. doi:10.1111/coa.13177

202. Chatham CH, Taylor KI, Charman T, et al. Adaptive behavior in autism: Minimal clinically important differences on the Vineland-II. *Autism Res*. Feb 2018;11(2):270-283. doi:10.1002/aur.1874

203. Yuksel S, Ayhan S, Nabiyev V, et al. Minimum clinically important difference of the health-related quality of life scales in adult spinal deformity calculated by latent class analysis: is it appropriate to use the same values for surgical and nonsurgical patients? *Spine J*. Jan 2019;19(1):71-78. doi:10.1016/j.spinee.2018.07.005

204. Brigden A, Parslow RM, Gaunt D, Collin SM, Jones A, Crawley E. Defining the minimally clinically important difference of the SF-36 physical function subscale for paediatric CFS/ME: triangulation using three different methods. *Health Qual Life Outcomes*. Oct 19 2018;16(1):202. doi:10.1186/s12955-018-1028-2

205. Asher AL, Kerezoudis P, Mummaneni PV, et al. Defining the minimum clinically important difference for grade I degenerative lumbar spondylolisthesis: insights from the Quality Outcomes Database. *Neurosurg Focus*. Jan 2018;44(1):E2. doi:10.3171/2017.10.Focus17554

206. Kerezoudis P, Yost KJ, Tombers NM, Celda MP, Carlson ML, Link MJ. Defining the Minimal Clinically Important Difference for Patients With Vestibular Schwannoma: Are all Quality-of-Life Scores Significant? *Neurosurgery*. Dec 1 2019;85(6):779-785. doi:10.1093/neuros/nyy467

207. Sutton RM, McDonald EL, Shakked RJ, Fuchs D, Raikin SM. Determination of Minimum Clinically Important Difference (MCID) in Visual Analog Scale (VAS) Pain and Foot and Ankle Ability Measure (FAAM) Scores After Hallux Valgus Surgery. *Foot Ankle Int*. Jun 2019;40(6):687-693. doi:10.1177/1071100719834539

208. Gowd AK, Cvetanovich GL, Liu JN, et al. Preoperative Mental Health Scores and Achieving Patient Acceptable Symptom State Are Predictive of Return to Work After Arthroscopic Rotator Cuff Repair. *Orthop J Sports Med*. Oct 2019;7(10):2325967119878415. doi:10.1177/2325967119878415

209. Park I, Oh MJ, Shin SJ. Minimal Clinically Important Differences and Correlating Factors for the Rowe Score and the American Shoulder and Elbow Surgeons Score After Arthroscopic Stabilization Surgery for Anterior Shoulder Instability. *Arthroscopy*. Jan 2019;35(1):54-59. doi:10.1016/j.arthro.2018.08.005

210. Deutscher D, Cook KF, Kallen MA, et al. Clinical Interpretation of the Neck Functional Status Computerized Adaptive Test. *J Orthop Sports Phys Ther*. Dec 2019;49(12):875-886. doi:10.2519/jospt.2019.8862

211. Alma HJ, de Jong C, Jelusic D, et al. Thresholds for clinically important deterioration versus improvement in COPD health status: results from a randomised controlled trial in pulmonary rehabilitation and an observational study during routine clinical practice. *BMJ Open*. Jun 28 2019;9(6):e025776. doi:10.1136/bmjopen-2018-025776

212. Trøstrup J, Andersen H, Kam CAM, Magnusson SP, Beyer N. Assessment of Mobility in Older People Hospitalized for Medical Illness Using the de Morton Mobility Index and Cumulated Ambulation Score-Validity and Minimal Clinical Important Difference. *J Geriatr Phys Ther*. Jul/Sep 2019;42(3):153-160. doi:10.1519/jpt.0000000000000170

213. Stacy M, Sajatovic M, Kane JM, et al. Abnormal involuntary movement scale in tardive dyskinesia: Minimal clinically important difference. *Mov Disord*. Aug 2019;34(8):1203-1209. doi:10.1002/mds.27769

214. Gerardo CJ, Vissoci JRN, de Oliveira LP, et al. The validity, reliability and minimal clinically important difference of the patient specific functional scale in snake envenomation. *PLoS One*. 2019;14(3):e0213077. doi:10.1371/journal.pone.0213077

215. Hiragami S, Inoue Y, Harada K. Minimal clinically important difference for the Fugl-Meyer assessment of the upper extremity in convalescent stroke patients with moderate to severe hemiparesis. *J Phys Ther Sci*. Nov 2019;31(11):917-921. doi:10.1589/jpts.31.917

216. Xue WW, Zhang P, Zou HD. Responsiveness and minimal clinically important difference of the Chinese version of the Low Vision Quality of Life Questionnaire after cataract surgery. *Int J Ophthalmol*. 2019;12(3):504-509. doi:10.18240/ijo.2019.03.23

217. Kato S, Oshima Y, Matsubayashi Y, Taniguchi Y, Tanaka S, Takeshita K. Minimum clinically important difference in outcome scores among patients undergoing cervical laminoplasty. *Eur Spine J*. May 2019;28(5):1234-1241. doi:10.1007/s00586-019-05945-y

218. Benaim C, Blaser S, Léger B, Vuistiner P, Luthi F. "Minimal clinically important difference" estimates of 6 commonly-used performance tests in patients with chronic musculoskeletal pain completing a work-related multidisciplinary rehabilitation program. *BMC Musculoskelet Disord*. Jan 5 2019;20(1):16. doi:10.1186/s12891-018-2382-2

219. Kato S, Oshima Y, Matsubayashi Y, Taniguchi Y, Tanaka S, Takeshita K. Minimum Clinically Important Difference and Patient Acceptable Symptom State of Japanese Orthopaedic Association Score in Degenerative Cervical Myelopathy Patients. *Spine (Phila Pa 1976)*. May 15 2019;44(10):691-697. doi:10.1097/brs.0000000000002928

220. Tashjian RZ, Shin J, Broschinsky K, et al. Minimal clinically important differences in the American Shoulder and Elbow Surgeons, Simple Shoulder Test, and visual analog scale pain scores after arthroscopic rotator cuff repair. *J Shoulder Elbow Surg*. Jul 2020;29(7):1406-1411. doi:10.1016/j.jse.2019.11.018

221. Cvetanovich GL, Gowd AK, Liu JN, et al. Establishing clinically significant outcome after arthroscopic rotator cuff repair. *J Shoulder Elbow Surg*. May 2019;28(5):939-948. doi:10.1016/j.jse.2018.10.013

222. Tantilipikorn P, Saisombat P, Phonpornpaiboon P, Pinkaew B, Lermankul W, Bunnag C. Minimal clinically important difference for the rhinoconjunctivitis quality of life questionnaire in allergic rhinitis in Thai population. *Asia Pac Allergy*. Jan 2019;9(1):e6. doi:10.5415/apallergy.2019.9.e6

223. Spina E, Topa A, Iodice R, et al. Six-minute walk test is reliable and sensitive in detecting response to therapy in CIDP. *J Neurol*. Apr 2019;266(4):860-865. doi:10.1007/s00415-019-09207-1

224. Sandvall B, Okoroafor UC, Gerull W, Guattery J, Calfee RP. Minimal Clinically Important Difference for PROMIS Physical Function in Patients With Distal Radius Fractures. *J Hand Surg Am*. Jun 2019;44(6):454-459.e1. doi:10.1016/j.jhsa.2019.02.015

225. Kandathil CK, Saltychev M, Abdelwahab M, Spataro EA, Moubayed SP, Most SP. Minimal Clinically Important Difference of the Standardized Cosmesis and Health Nasal Outcomes Survey. *Aesthet Surg J*. Jul 12 2019;39(8):837-840. doi:10.1093/asj/sjz070

226. Gowd AK, Lalehzarian SP, Liu JN, et al. Factors Associated With Clinically Significant Patient-Reported Outcomes After Primary Arthroscopic Partial Meniscectomy. *Arthroscopy*. May 2019;35(5):1567-1575.e3. doi:10.1016/j.arthro.2018.12.014

227. Clement ND, Weir D, Holland J, Gerrand C, Deehan DJ. Meaningful changes in the Short Form 12 physical and mental summary scores after total knee arthroplasty. *Knee*. Aug 2019;26(4):861-868. doi:10.1016/j.knee.2019.04.018

228. van Munster CE, Kaya L, Obura M, Kalkers NF, Uitdehaag BM. Minimal clinically important difference of improvement on the Arm Function in Multiple Sclerosis Questionnaire (AMSQ). *Mult Scler*. Apr 2020;26(4):505-508. doi:10.1177/1352458518823489

229. Andrews JS, Desai U, Kirson NY, Zichlin ML, Ball DE, Matthews BR. Disease severity and minimal clinically important differences in clinical outcome assessments for Alzheimer's disease clinical trials. *Alzheimers Dement (N Y)*. 2019;5:354-363. doi:10.1016/j.trci.2019.06.005

230. Ogura K, Uehara K, Akiyama T, et al. Minimal clinically important differences in Toronto Extremity Salvage Score for patients with lower extremity sarcoma. *J Orthop Sci*. Mar 2020;25(2):315-318. doi:10.1016/j.jos.2019.03.022

231. Gowd AK, Charles MD, Liu JN, et al. Single Assessment Numeric Evaluation (SANE) is a reliable metric to measure clinically significant improvements following shoulder arthroplasty. *J Shoulder Elbow Surg*. Nov 2019;28(11):2238-2246. doi:10.1016/j.jse.2019.04.041

232. Hung M, Baumhauer JF, Licari FW, Voss MW, Bounsanga J, Saltzman CL. PROMIS and FAAM Minimal Clinically Important Differences in Foot and Ankle Orthopedics. *Foot Ankle Int*. Jan 2019;40(1):65-73. doi:10.1177/1071100718800304

233. Louwerens JKG, van den Bekerom MPJ, van Royen BJ, Eygendaal D, van Noort A, Sierevelt IN. Quantifying the minimal and substantial clinical benefit of the Constant-Murley score and the Disabilities of the Arm, Shoulder and Hand score in patients with calcific tendinitis of the rotator cuff. *JSES Int*. Sep 2020;4(3):606-611. doi:10.1016/j.jseint.2020.05.001

234. Kuhns BD, Reuter J, Lawton D, Kenney RJ, Baumhauer JF, Giordano BD. Threshold Values for Success After Hip Arthroscopy Using the Patient-Reported Outcomes Measurement Information System Assessment: Determining the Minimum Clinically Important Difference and Patient Acceptable Symptomatic State. *Am J Sports Med*. Nov 2020;48(13):3280-3287. doi:10.1177/0363546520960461

235. Sobreira M, Almeida MP, Gomes A, Lucas M, Oliveira A, Marques A. Minimal Clinically Important Differences for Measures of Pain, Lung Function, Fatigue, and Functionality in Spinal Cord Injury. *Phys Ther*. Feb 4 2021;101(2)doi:10.1093/ptj/pzaa210

236. Okoroha KR, Lu Y, Nwachukwu BU, et al. How Should We Define Clinically Significant Improvement on Patient-Reported Outcomes Measurement Information System Test for Patients Undergoing Knee Meniscal Surgery? *Arthroscopy*. Jan 2020;36(1):241-250. doi:10.1016/j.arthro.2019.07.036

237. Kuo AC, Giori NJ, Bowe TR, et al. Comparing Methods to Determine the Minimal Clinically Important Differences in Patient-Reported Outcome Measures for Veterans Undergoing Elective Total Hip or Knee Arthroplasty in Veterans Health Administration Hospitals. *JAMA Surg*. May 1 2020;155(5):404-411. doi:10.1001/jamasurg.2020.0024

238. Butler J, Khan MS, Mori C, et al. Minimal clinically important difference in quality of life scores for patients with heart failure and reduced ejection fraction. *Eur J Heart Fail*. Jun 2020;22(6):999-1005. doi:10.1002/ejhf.1810

239. Ekström MP, Bornefalk H, Sköld CM, et al. Minimal Clinically Important Differences and Feasibility of Dyspnea-12 and the Multidimensional Dyspnea Profile in Cardiorespiratory Disease. *J Pain Symptom Manage*. Nov 2020;60(5):968-975.e1. doi:10.1016/j.jpainsymman.2020.05.028

240. Franchignoni F, Ferriero G, Giordano A, Monticone M, Grioni G, Burger H. The minimal clinically-important difference of the Prosthesis Evaluation Questionnaire - Mobility Scale in subjects undergoing lower limb prosthetic rehabilitation training. *Eur J Phys Rehabil Med*. Feb 2020;56(1):82-87. doi:10.23736/s1973-9087.19.05799-x

241. Goudman L, Smedt A, Forget P, Moens M. Determining the Minimal Clinical Important Difference for Medication Quantification Scale III and Morphine Milligram Equivalents in Patients with Failed Back Surgery Syndrome. *J Clin Med*. Nov 21 2020;9(11)doi:10.3390/jcm9113747

242. Jacquet C, Pioger C, Khakha R, et al. Evaluation of the "Minimal Clinically Important Difference" (MCID) of the KOOS, KSS and SF-12 scores after open-wedge high tibial osteotomy. *Knee Surg Sports Traumatol Arthrosc*. Mar 2021;29(3):820-826. doi:10.1007/s00167-020-06026-0

243. Ogura T, Ackermann J, Barbieri Mestriner A, Merkely G, Gomoll AH. Minimal Clinically Important Differences and Substantial Clinical Benefit in Patient-Reported Outcome Measures after Autologous Chondrocyte Implantation. *Cartilage*. Oct 2020;11(4):412-422. doi:10.1177/1947603518799839

244. Lewandrowski KU, PST DEC, P DEC, Yeung A. Minimal Clinically Important Difference in Patient-Reported Outcome Measures with the Transforaminal Endoscopic Decompression for Lateral Recess and Foraminal Stenosis. *Int J Spine Surg*. Apr 2020;14(2):254-266. doi:10.14444/7034

245. Roux P, Brunet-Gouet E, Ehrminger M, et al. Minimum clinically important differences for the Functioning Assessment Short Test and a battery of neuropsychological tests in bipolar disorders: results from the FACE-BD cohort. *Epidemiol Psychiatr Sci*. Jul 20 2020;29:e144. doi:10.1017/s2045796020000566

246. Carton P, Filan D. Defining the Minimal Clinically Important Difference in Athletes Undergoing Arthroscopic Correction of Sports-Related Femoroacetabular Impingement: The Percentage of Possible Improvement. *Orthop J Sports Med*. Jan 2020;8(1):2325967119894747. doi:10.1177/2325967119894747

247. Ekström M, Johnson MJ, Huang C, Currow DC. Minimal clinically important differences in average, best, worst and current intensity and unpleasantness of chronic breathlessness. *Eur Respir J*. Aug 2020;56(2)doi:10.1183/13993003.02202-2019

248. Shulman MA, Kasza J, Myles PS. Defining the Minimal Clinically Important Difference and Patient-acceptable Symptom State Score for Disability Assessment in Surgical Patients. *Anesthesiology*. Jun 2020;132(6):1362-1370. doi:10.1097/aln.0000000000003240

249. Okano I, Ortiz Miller C, Salzmann SN, et al. Minimum Clinically Important Differences of the Hospital for Special Surgery Dysphagia and Dysphonia Inventory and Other Dysphagia Measurements in Patients Undergoing ACDF. *Clin Orthop Relat Res*. Oct 2020;478(10):2309-2320. doi:10.1097/corr.0000000000001236

250. Lizaur-Utrilla A, Gonzalez-Parreño S, Martinez-Mendez D, Miralles-Muñoz FA, Lopez-Prats FA. Minimal clinically important differences and substantial clinical benefits for Knee Society Scores. *Knee Surg Sports Traumatol Arthrosc*. May 2020;28(5):1473-1478. doi:10.1007/s00167-019-05543-x

251. Ogura K, Bartelstein MK, Yakoub MA, Nikolic Z, Boland PJ, Healey JH. Minimal clinically important differences in SF-36 global score: Current value in orthopedic oncology. *J Orthop Res*. Oct 2021;39(10):2116-2123. doi:10.1002/jor.24944

252. Kazmers NH, Qiu Y, Yoo M, Stephens AR, Tyser AR, Zhang Y. The Minimal Clinically Important Difference of the PROMIS and QuickDASH Instruments in a Nonshoulder Hand and Upper Extremity Patient Population. *J Hand Surg Am*. May 2020;45(5):399-407.e6. doi:10.1016/j.jhsa.2019.12.002

253. Vander Mijnsbrugge GJ, Molenaar C, Buyl R, Westert G, van der Wees PJ. How is your proctology patient really doing? Outcome measurement in proctology: development, design and validation study of the Proctoprom. *Tech Coloproctol*. Apr 2020;24(4):291-300. doi:10.1007/s10151-020-02156-2

254. Haunschild ED, Gilat R, Fu MC, et al. Establishing the Minimal Clinically Important Difference, Patient Acceptable Symptomatic State, and Substantial Clinical Benefit of the PROMIS Upper Extremity Questionnaire After Rotator Cuff Repair. *Am J Sports Med*. Dec 2020;48(14):3439-3446. doi:10.1177/0363546520964957

255. Nelson EO, Kliethermes S, Heiderscheit B. Construct Validity and Responsiveness of the University of Wisconsin Running Injury and Recovery Index. *J Orthop Sports Phys Ther*. Dec 2020;50(12):702-710. doi:10.2519/jospt.2020.9698

256. Calixtre LB, Oliveira AB, Alburquerque-Sendín F, Armijo-Olivo S. What is the minimal important difference of pain intensity, mandibular function, and headache impact in patients with temporomandibular disorders? Clinical significance analysis of a randomized controlled trial. *Musculoskelet Sci Pract*. Apr 2020;46:102108. doi:10.1016/j.msksp.2020.102108

257. McLawhorn AS, Buller LT, Blevins JL, Lee YY, Su EP. What Are the Benefits of Hip Resurfacing in Appropriate Patients? A Retrospective, Propensity Score-Matched Analysis. *Hss j*. Dec 2020;16(Suppl 2):316-326. doi:10.1007/s11420-019-09729-4

258. Yao M, Xu BP, Li ZJ, et al. A comparison between the low back pain scales for patients with lumbar disc herniation: validity, reliability, and responsiveness. *Health Qual Life Outcomes*. Jun 10 2020;18(1):175. doi:10.1186/s12955-020-01403-2

259. Vanhorn TA, Knio ZO, O'Gara TJ. Defining a Minimum Clinically Important Difference in Patient-Reported Outcome Measures in Lumbar Tubular Microdecompression Patients. *Int J Spine Surg*. Aug 2020;14(4):538-543. doi:10.14444/7071

260. Bächinger D, Mlynski R, Weiss NM. Establishing the minimal clinically important difference (MCID) of the Zurich Chronic Middle Ear Inventory (ZCMEI-21) in patients treated for chronic middle ear disease. *Eur Arch Otorhinolaryngol*. Apr 2020;277(4):1039-1044. doi:10.1007/s00405-020-05819-w

261. Hu X, Jing M, Zhang M, Yang P, Yan X. Responsiveness and minimal clinically important difference of the EQ-5D-5L in cervical intraepithelial neoplasia: a longitudinal study. *Health Qual Life Outcomes*. Oct 2 2020;18(1):324. doi:10.1186/s12955-020-01578-8

262. Monticone M, Frigau L, Vernon H, et al. Reliability, responsiveness and minimal clinically important difference of the two Fear Avoidance and Beliefs Questionnaire scales in Italian subjects with chronic low back pain undergoing multidisciplinary rehabilitation. *Eur J Phys Rehabil Med*. Oct 2020;56(5):600-606. doi:10.23736/s1973-9087.20.06158-4

263. Gray AJ, Huston M, Didericksen D, Meyer TK, Merati A, Brisebois S. The minimal clinically important difference of the dyspnea index in laryngotracheal stenosis. *Laryngoscope*. Jul 2020;130(7):1775-1779. doi:10.1002/lary.28331

264. Burgstaller JM, Wertli MM, Ulrich NH, et al. Evaluating the Minimal Clinically Important Difference of EQ-5D-3L in Patients With Degenerative Lumbar Spinal Stenosis: A Swiss Prospective Multicenter Cohort Study. *Spine (Phila Pa 1976)*. Sep 15 2020;45(18):1309-1316. doi:10.1097/brs.0000000000003501

265. Pintér D, Janszky J, Kovács N. Minimal Clinically Important Differences for Burke-Fahn-Marsden Dystonia Rating Scale and 36-Item Short-Form Health Survey. *Mov Disord*. Jul 2020;35(7):1218-1223. doi:10.1002/mds.28057

266. Chen Haisi, Huang Jinhai, Gao Rongrong, Ye Junming, Chen Min, Wang Qinmei. Application of Chinese VF-11R questionnaire in presenting low vision cataract patients undergoing surgery. *Chin J Exp Ophthalmol,*. 2020;38(4):6.

267. Chen Haisi, Tang Nana, Zhu Miaomiao, et al. Minimum clinically important difference of Catquest 9SF-CN scale. *Chinese Journal of optometry and Vision Science*. 2020;22(3):6.

268. HU Xin. *The measurement characteristics and MCID of the EQ-5D-5L questionnaire in patients with cervical precancerous lesions*. Shihezi University; 2020.

269. YANG Xue, JIANG Qiao, XU Chi, FU Jun, LI Rui, CHEN  Jiying. Minimal clinically important difference for HSS of patients with primary osteoarthritis after total knee arthroplasty. 2020;

270. Kim MS, Koh IJ, Choi KY, et al. The Minimal Clinically Important Difference (MCID) for the WOMAC and Factors Related to Achievement of the MCID After Medial Opening Wedge High Tibial Osteotomy for Knee Osteoarthritis. *Am J Sports Med*. Jul 2021;49(9):2406-2415. doi:10.1177/03635465211016853

271. Haase I, Kladny B. Clinical Relevance of Changes in Pain Intensity in Patients with Specific Back Pain. *Z Orthop Unfall*. Apr 2022;160(2):213-221. Klinische Bedeutung von Veränderungen der Schmerzstärke bei Patienten mit spezifischen Rückenschmerzen. doi:10.1055/a-1304-3677

272. Bongers MER, Groot OQ, Thio Q, et al. Prospective study for establishing minimal clinically important differences in patients with surgery for lower extremity metastases. *Acta Oncol*. Jun 2021;60(6):714-720. doi:10.1080/0284186x.2021.1890333

273. Puchar A, Panel P, Oppenheimer A, Du Cheyron J, Fritel X, Fauconnier A. The ENDOPAIN 4D Questionnaire: A New Validated Tool for Assessing Pain in Endometriosis. *J Clin Med*. Jul 21 2021;10(15)doi:10.3390/jcm10153216

274. Nakarai H, Kato S, Kawamura N, et al. Minimal clinically important difference in patients who underwent decompression alone for lumbar degenerative disease. *Spine J*. Apr 2022;22(4):549-560. doi:10.1016/j.spinee.2021.10.010

275. Pruijssers B, van der Vaart L, Milani F, Roovers JP, Vollebregt A, van der Vaart H. Minimal Clinically Important Difference (MCID) for the Pelvic Organ Prolapse-Urinary Incontinence Sexual Function Questionnaire - IUGA Revised (PISQ-IR). *J Sex Med*. Jul 2021;18(7):1265-1270. doi:10.1016/j.jsxm.2021.04.005

276. Kawakami D, Fujitani S, Morimoto T, et al. Prevalence of post-intensive care syndrome among Japanese intensive care unit patients: a prospective, multicenter, observational J-PICS study. *Crit Care*. Feb 16 2021;25(1):69. doi:10.1186/s13054-021-03501-z

277. Gibson CJ, Huang AJ, Larson JC, et al. Patient-centered change in the day-to-day impact of postmenopausal vaginal symptoms: results from a multicenter randomized trial. *Am J Obstet Gynecol*. Jul 2020;223(1):99.e1-99.e9. doi:10.1016/j.ajog.2019.12.270

278. Rosinsky PJ, Kyin C, Maldonado DR, et al. Determining Clinically Meaningful Thresholds for the Nonarthritic Hip Score in Patients Undergoing Arthroscopy for Femoroacetabular Impingement Syndrome. *Arthroscopy*. Oct 2021;37(10):3113-3121. doi:10.1016/j.arthro.2021.03.059

279. Vishwanathan K, Braithwaite I. Construct validity and responsiveness of commonly used patient reported outcome instruments in decompression for lumbar spinal stenosis. *J Clin Orthop Trauma*. May 2021;16:125-131. doi:10.1016/j.jcot.2021.01.002

280. Tack J, Camilleri M, Hale M, et al. Establishing Minimal Clinically Important Differences in Quality of Life Measures in Opioid-Induced Constipation. *Clin Gastroenterol Hepatol*. Apr 2022;20(4):855-863. doi:10.1016/j.cgh.2021.05.004

281. Malavolta EA, Yamamoto GJ, Bussius DT, et al. Establishing minimal clinically important difference for the UCLA and ASES scores after rotator cuff repair. *Orthop Traumatol Surg Res*. Apr 2022;108(2):102894. doi:10.1016/j.otsr.2021.102894

282. Carlesso C, Piva SR, Smith C, Ammendolia C, Schneider MJ. Responsiveness of Outcome Measures in Nonsurgical Patients with Lumbar Spinal Stenosis: A Secondary Analysis From a Randomized Controlled Trial. *Spine (Phila Pa 1976)*. Jun 15 2021;46(12):788-795. doi:10.1097/brs.0000000000003920

283. Ogura T, Ackermann J, Mestriner AB, Merkely G, Gomoll AH. The Minimal Clinically Important Difference and Substantial Clinical Benefit in the Patient-Reported Outcome Measures of Patients Undergoing Osteochondral Allograft Transplantation in the Knee. *Cartilage*. Jan 2021;12(1):42-50. doi:10.1177/1947603518812552

284. Agustín RM, Crisostomo MJ, Sánchez-Martínez MP, Medina-Mirapeix F. Responsiveness and Minimal Clinically Important Difference of the Five Times Sit-to-Stand Test in Patients with Stroke. *Int J Environ Res Public Health*. Feb 26 2021;18(5)doi:10.3390/ijerph18052314

285. Nyring MRK, Olsen BS, Amundsen A, Rasmussen JV. Minimal Clinically Important Differences (MCID) for the Western Ontario Osteoarthritis of the Shoulder Index (WOOS) and the Oxford Shoulder Score (OSS). *Patient Relat Outcome Meas*. 2021;12:299-306. doi:10.2147/prom.S316920

286. Kim JW, Clark A, Birring SS, Atkins C, Whyte M, Wilson AM. Psychometric properties of patient reported outcome measures in idiopathic pulmonary fibrosis. *Chron Respir Dis*. Jan-Dec 2021;18:14799731211033925. doi:10.1177/14799731211033925

287. Bunclark K, Doughty N, Michael A, et al. A minimal clinically important difference measured by the Cambridge Pulmonary Hypertension Outcome Review for patients with idiopathic pulmonary arterial hypertension. *Pulm Circ*. Apr-Jun 2021;11(2):2045894021995055. doi:10.1177/2045894021995055

288. Sun Z, Li J, Luo G, Wang F, Hu Y, Fan C. What constitutes a clinically important change in Mayo Elbow Performance Index and range of movement after open elbow arthrolysis? *Bone Joint J*. Feb 2021;103-b(2):366-372. doi:10.1302/0301-620x.103b2.Bjj-2020-0259.R3

289. Lee DJ, Calfee RP. The Minimal Clinically Important Difference for PROMIS Physical Function in Patients With Thumb Carpometacarpal Arthritis. *Hand (N Y)*. Sep 2021;16(5):638-643. doi:10.1177/1558944719880025

290. Sabourin S, Tram J, Sheldon BL, Pilitsis JG. Defining minimal clinically important differences in pain and disability outcomes of patients with chronic pain treated with spinal cord stimulation. *J Neurosurg Spine*. Jun 4 2021:1-8. doi:10.3171/2020.11.Spine201431

291. Baker JF, Katz P, Michaud K. Defining Minimum Clinically Important Changes for the Patient Activity Scale II. *Arthritis Care Res (Hoboken)*. Oct 2021;73(10):1456-1460. doi:10.1002/acr.24335

292. Gordon D, Pines Y, Ben-Ari E, et al. Minimal clinically important difference, substantial clinical benefit, and patient acceptable symptom state of PROMIS upper extremity after total shoulder arthroplasty. *JSES Int*. Sep 2021;5(5):894-899. doi:10.1016/j.jseint.2021.05.003

293. Neela N, Olavarria OA, Rondon AP, et al. Validation of the minimal clinically important difference for modified activities assessment scale. *Am J Surg*. Apr 2022;223(4):770-773. doi:10.1016/j.amjsurg.2021.07.042

294. Filan D, Carton P. Chronic Hip Injury Has a Negative Emotional Impact on the Male Athlete With Femoroacetabular Impingement. *Arthroscopy*. Feb 2021;37(2):566-576. doi:10.1016/j.arthro.2020.10.035

295. Kazmers NH, Qiu Y, Ou Z, Presson AP, Tyser AR, Zhang Y. Minimal Clinically Important Difference of the PROMIS Upper-Extremity Computer Adaptive Test and QuickDASH for Ligament Reconstruction Tendon Interposition Patients. *J Hand Surg Am*. Jun 2021;46(6):516-516.e7. doi:10.1016/j.jhsa.2020.11.007

296. Ogura K, Yakoub MA, Christ AB, et al. The critical difference in the DASH (Disabilities of the Arm, Shoulder, and Hand) outcome measure after essential upper extremity tumor surgery. *J Shoulder Elbow Surg*. Sep 2021;30(9):e602-e609. doi:10.1016/j.jse.2020.11.027

297. Longo UG, De Salvatore S, Candela V, et al. Unicompartmental Knee Arthroplasty: Minimal Important Difference and Patient Acceptable Symptom State for the Forgotten Joint Score. *Medicina (Kaunas)*. Apr 1 2021;57(4)doi:10.3390/medicina57040324

298. Khor S, Flum DR, Strate LL, et al. Establishing Clinically Significant Patient-reported Outcomes for Diverticular Disease. *J Surg Res*. Aug 2021;264:20-29. doi:10.1016/j.jss.2021.01.045

299. Christensen MC, Fagiolini A, Florea I, Loft H, Cuomo A, Goodwin GM. Validation of the Oxford Depression Questionnaire: Sensitivity to change, minimal clinically important difference, and response threshold for the assessment of emotional blunting. *J Affect Disord*. Nov 1 2021;294:924-931. doi:10.1016/j.jad.2021.07.099

300. Ohno K, Tomori K, Sawada T, Kobayashi R. Examining minimal important change of the Canadian Occupational Performance Measure for subacute rehabilitation hospital inpatients. *J Patient Rep Outcomes*. Dec 20 2021;5(1):133. doi:10.1186/s41687-021-00405-y

301. Eckhard L, Munir S, Wood D, et al. Minimal important change and minimum clinically important difference values of the KOOS-12 after total knee arthroplasty. *Knee*. Mar 2021;29:541-546. doi:10.1016/j.knee.2021.03.005

302. Guo Shengxuan, Husiyuan, Guang Junxiu, et al. Minimal clinically important difference of Canadian Acute Respiratory Illnessand Flu Scale Chinese version in children with common cold. *Acad J Chin PLA Med Sch*. 2021;44(5):6.

303. Boekel I, Dutmer AL, Schiphorst Preuper HR, Reneman MF. Validation of the work ability index-single item and the pain disability index-work item in patients with chronic low back pain. *Eur Spine J*. Apr 2022;31(4):943-952. doi:10.1007/s00586-022-07109-x

304. Molino J, Harrington J, Racine-Avila J, Aaron R. Deconstructing the Minimum Clinically Important Difference (MCID). *Orthop Res Rev*. 2022;14:35-42. doi:10.2147/orr.S349268

305. Foong WS, Zeng GJ, Goh GS, Hao Y, Lie DTT, Chang PCC. Determining the Minimal Clinically Important Difference on the Oxford Shoulder Instability Score in Patients Undergoing Arthroscopic Bankart Repair for Shoulder Instability. *Orthop J Sports Med*. Jan 2022;10(1):23259671211060023. doi:10.1177/23259671211060023

306. Wong K, Zeng L, Zhang L, et al. Minimal clinically important differences in the brief pain inventory in patients with bone metastases. *Support Care Cancer*. Jul 2013;21(7):1893-9. doi:10.1007/s00520-013-1731-9

307. Nathan SD, du Bois RM, Albera C, et al. Validation of test performance characteristics and minimal clinically important difference of the 6-minute walk test in patients with idiopathic pulmonary fibrosis. *Respir Med*. Jul 2015;109(7):914-22. doi:10.1016/j.rmed.2015.04.008

308. Carse B, Scott H, Davie-Smith F, Brady L, Colvin J. Minimal clinically important difference in walking velocity, gait profile score and two minute walk test for individuals with lower limb amputation. *Gait Posture*. Jul 2021;88:221-224. doi:10.1016/j.gaitpost.2021.06.001

309. YanG Lihong. Development of minimal clinically important difference for proteinuria in adult with diabetic kidneydisease. Guangzhou University of Chinese Medicine; 2020.

310. Cella D, Eton DT, Fairclough DL, et al. What is a clinically meaningful change on the Functional Assessment of Cancer Therapy-Lung (FACT-L) Questionnaire? Results from Eastern Cooperative Oncology Group (ECOG) Study 5592. *J Clin Epidemiol*. Mar 2002;55(3):285-95. doi:10.1016/s0895-4356(01)00477-2

311. Shulman LM, Gruber-Baldini AL, Anderson KE, Fishman PS, Reich SG, Weiner WJ. The clinically important difference on the unified Parkinson's disease rating scale. *Arch Neurol*. Jan 2010;67(1):64-70. doi:10.1001/archneurol.2009.295

312. Luo Xiaoxia. *Study on the minimum clinically important difference of health status measurement instrument for cardiac insufficiency*. Beijing University of Chinese Medicine; 2010.

313. Parker SL, Adogwa O, Paul AR, et al. Utility of minimum clinically important difference in assessing pain, disability, and health state after transforaminal lumbar interbody fusion for degenerative lumbar spondylolisthesis. *J Neurosurg Spine*. May 2011;14(5):598-604. doi:10.3171/2010.12.Spine10472

314. Parker SL, Mendenhall SK, Shau D, et al. Determination of minimum clinically important difference in pain, disability, and quality of life after extension of fusion for adjacent-segment disease. *J Neurosurg Spine*. Jan 2012;16(1):61-7. doi:10.3171/2011.8.Spine1194

315. Patel AS, Siegert RJ, Keir GJ, et al. The minimal important difference of the King's Brief Interstitial Lung Disease Questionnaire (K-BILD) and forced vital capacity in interstitial lung disease. *Respir Med*. Sep 2013;107(9):1438-43. doi:10.1016/j.rmed.2013.06.009

316. Le QA, Doctor JN, Zoellner LA, Feeny NC. Minimal clinically important differences for the EQ-5D and QWB-SA in Post-traumatic Stress Disorder (PTSD): results from a Doubly Randomized Preference Trial (DRPT). *Health Qual Life Outcomes*. Apr 12 2013;11:59. doi:10.1186/1477-7525-11-59

317. Kon SS, Canavan JL, Nolan CM, et al. The 4-metre gait speed in COPD: responsiveness and minimal clinically important difference. *Eur Respir J*. May 2014;43(5):1298-305. doi:10.1183/09031936.00088113

318. Carlson ML, Tveiten Ø V, Yost KJ, Lohse CM, Lund-Johansen M, Link MJ. The Minimal Clinically Important Difference in Vestibular Schwannoma Quality-of-Life Assessment: An Important Step beyond P < .05. *Otolaryngol Head Neck Surg*. Aug 2015;153(2):202-8. doi:10.1177/0194599815585508

319. Wiegersma M, Panman CM, Berger MY, De Vet HC, Kollen BJ, Dekker JH. Minimal important change in the pelvic floor distress inventory-20 among women opting for conservative prolapse treatment. *Am J Obstet Gynecol*. Apr 2017;216(4):397.e1-397.e7. doi:10.1016/j.ajog.2016.10.010

320. Pandian S, Arya KN, Kumar D. Minimal clinically important difference of the lower-extremity fugl-meyer assessment in chronic-stroke. *Top Stroke Rehabil*. Aug 2016;23(4):233-9. doi:10.1179/1945511915y.0000000003

321. Lee WC, Kwan YH, Chong HC, Yeo SJ. The minimal clinically important difference for Knee Society Clinical Rating System after total knee arthroplasty for primary osteoarthritis. *Knee Surg Sports Traumatol Arthrosc*. Nov 2017;25(11):3354-3359. doi:10.1007/s00167-016-4208-9

322. Hur SA, Guler SA, Khalil N, et al. Minimal Important Difference for Physical Activity and Validity of the International Physical Activity Questionnaire in Interstitial Lung Disease. *Ann Am Thorac Soc*. Jan 2019;16(1):107-115. doi:10.1513/AnnalsATS.201804-265OC

323. Humphries SM, Swigris JJ, Brown KK, et al. Quantitative high-resolution computed tomography fibrosis score: performance characteristics in idiopathic pulmonary fibrosis. *Eur Respir J*. Sep 2018;52(3)doi:10.1183/13993003.01384-2018

324. Guzik A, Drużbicki M, Wolan-Nieroda A, Przysada G, Kwolek A. The Wisconsin gait scale - The minimal clinically important difference. *Gait Posture*. Feb 2019;68:453-457. doi:10.1016/j.gaitpost.2018.12.036

325. Suzuki A, Kondoh Y, Swigris JJ, et al. Performance of the COPD Assessment Test in patients with connective tissue disease-associated interstitial lung disease. *Respir Med*. Apr 2019;150:15-20. doi:10.1016/j.rmed.2019.01.017

326. Wu JJ, Lin C, Sun L, et al. Minimal clinically important difference (MCID) for work productivity and activity impairment (WPAI) questionnaire in psoriasis patients. *J Eur Acad Dermatol Venereol*. Feb 2019;33(2):318-324. doi:10.1111/jdv.15098

327. Guzik A, Drużbicki M, Wolan-Nieroda A, Turolla A, Kiper P. Estimating Minimal Clinically Important Differences for Knee Range of Motion after Stroke. *J Clin Med*. Oct 15 2020;9(10)doi:10.3390/jcm9103305

328. Godi M, Arcolin I, Giardini M, Corna S, Schieppati M. Responsiveness and minimal clinically important difference of the Mini-BESTest in patients with Parkinson's disease. *Gait Posture*. Jul 2020;80:14-19. doi:10.1016/j.gaitpost.2020.05.004

329. Bonnefoy-Mazure A, Lübbeke A, Miozzari HH, et al. Walking Speed and Maximal Knee Flexion During Gait After Total Knee Arthroplasty: Minimal Clinically Important Improvement Is Not Determinable; Patient Acceptable Symptom State Is Potentially Useful. *J Arthroplasty*. Oct 2020;35(10):2865-2871.e2. doi:10.1016/j.arth.2020.05.038

330. Rebelo P, Oliveira A, Andrade L, Valente C, Marques A. Minimal Clinically Important Differences for Patient-Reported Outcome Measures of Fatigue in Patients With COPD Following Pulmonary Rehabilitation. *Chest*. Aug 2020;158(2):550-561. doi:10.1016/j.chest.2020.02.045

331. Wynne SC, Patel S, Barker RE, et al. Anxiety and depression in bronchiectasis: Response to pulmonary rehabilitation and minimal clinically important difference of the Hospital Anxiety and Depression Scale. *Chron Respir Dis*. Jan-Dec 2020;17:1479973120933292. doi:10.1177/1479973120933292

332. Bae E, Choi SE, Lee H, Shin G, Kang D. Validity of EQ-5D utility index and minimal clinically important difference estimation among patients with chronic obstructive pulmonary disease. *BMC Pulm Med*. Mar 23 2020;20(1):73. doi:10.1186/s12890-020-1116-z

333. Saengsuwan J, Vichiansiri R. Minimal clinically important difference of Gait Assessment and Intervention Tool (GAIT) in patients with sub-acute stroke. *Eur J Phys Rehabil Med*. Dec 2021;57(6):874-878. doi:10.23736/s1973-9087.21.06735-6

334. Guzik A, Drużbicki M, Perenc L, Wolan-Nieroda A, Turolla A, Kiper P. Establishing the Minimal Clinically Important Differences for Sagittal Hip Range of Motion in Chronic Stroke Patients. *Front Neurol*. 2021;12:700190. doi:10.3389/fneur.2021.700190

335. Putman S, Dartus J, Migaud H, et al. Can the minimal clinically important difference be determined in a French-speaking population with primary hip replacement using one PROM item and the Anchor strategy? *Orthop Traumatol Surg Res*. May 2021;107(3):102830. doi:10.1016/j.otsr.2021.102830

336. Khow YZ, Liow MHL, Goh GS, Chen JY, Lo NN, Yeo SJ. The oxford knee score minimal clinically important difference for revision total knee arthroplasty. *Knee*. Oct 2021;32:211-217. doi:10.1016/j.knee.2021.08.020

337. Khow YZ, Liow MHL, Goh GS, Chen JY, Lo NN, Yeo SJ. Defining the minimal clinically important difference for the knee society score following revision total knee arthroplasty. *Knee Surg Sports Traumatol Arthrosc*. Jun 11 2021;doi:10.1007/s00167-021-06628-2

338. Mao F, Sun Y, Wang J, Huang Y, Lu Y, Cao F. Sensitivity to change and minimal clinically important difference of Edinburgh postnatal depression scale. *Asian J Psychiatr*. Dec 2021;66:102873. doi:10.1016/j.ajp.2021.102873

339. Haase I, Winkeler M, Imgart H. Ascertaining minimal clinically meaningful changes in symptoms of depression rated by the 15-item Centre for Epidemiologic Studies Depression Scale. *J Eval Clin Pract*. Jun 2022;28(3):500-506. doi:10.1111/jep.13629

340. Koopman JE, van Kooij YE, Selles RW, et al. Determining the Minimally Important Change of the Michigan Hand outcomes Questionnaire in patients undergoing trigger finger release. *J Hand Ther*. Jul 24 2021;doi:10.1016/j.jht.2021.06.003
